# GWarrange: a pre- and post- genome-wide association studies pipeline for detecting phenotype-associated genome rearrangement events

**DOI:** 10.1099/mgen.0.001268

**Published:** 2024-07-09

**Authors:** Yi Ling Tam, Sarah Cameron, Andrew Preston, Lauren Cowley

**Affiliations:** 1 The Milner Centre for Evolution and Department of Life Sciences, University of Bath, Claverton Down, Bath, BA2 7AY, UK

**Keywords:** bacterial genome-wide association studies, *Bordetella pertussis*, *Enterococcus faecium*, genome rearrangement, *k*-mers, repeat sequences

## Abstract

The use of *k*-mers to capture genetic variation in bacterial genome-wide association studies (bGWAS) has demonstrated its effectiveness in overcoming the plasticity of bacterial genomes by providing a comprehensive array of genetic variants in a genome set that is not confined to a single reference genome. However, little attempt has been made to interpret *k*-mers in the context of genome rearrangements, partly due to challenges in the exhaustive and high-throughput identification of genome structure and individual rearrangement events. Here, we present *GWarrange*, a pre- and post-bGWAS processing methodology that leverages the unique properties of *k*-mers to facilitate bGWAS for genome rearrangements. Repeat sequences are common instigators of genome rearrangements through intragenomic homologous recombination, and they are commonly found at rearrangement boundaries. Using whole-genome sequences, repeat sequences are replaced by short placeholder sequences, allowing the regions flanking repeats to be incorporated into relatively short *k*-mers. Then, locations of flanking regions in significant *k*-mers are mapped back to complete genome sequences to visualise genome rearrangements. Four case studies based on two bacterial species (*Bordetella pertussis* and *Enterococcus faecium*) and a simulated genome set are presented to demonstrate the ability to identify phenotype-associated rearrangements. *GWarrange* is available at https://github.com/DorothyTamYiLing/GWarrangehttps://github.com/DorothyTamYiLing/genome_rearrangement.git.

**Impact Statement**

Recently, microbiologists have been directing their attention towards genome rearrangements due to their ubiquity in bacterial genomes, their unknown roles in generating phenotypic variation, and the resulting evolutionary implications. With the growth of population-scale datasets and advances in long-read sequencing technology, routine evaluation of the impact of genome rearrangements on phenotype is increasingly becoming feasible. To overcome the challenges of high-throughput characterization of genome structure in large datasets, we present a novel method that provides association between genome rearrangements and phenotype, offering additional insights into host adaptation, niche adaptation, and pathogenicity. We hope this simple and easy-to-use pipeline, available as open-source software, will become integral to the wider microbial genomics research community interested in investigating genome rearrangement contributions to phenotype in their own datasets.

## Data Summary

Tables S1, S2, S3 and S4, available in the online version of this article. Closed genome sequences and simulated genome sequences used in each of the four examples.

## Introduction

Genome-wide association studies (GWAS) explore the relationship between genotype and phenotype by examining the association between numerous genetic variants across often large numbers of genomes alongside the variation observed in a particular phenotype [1]. In the past decades, it has been applied in the study of bacterial genomes [2,6]. One of the major advances in bacterial genome-wide association studies (bGWAS) in overcoming the plasticity of bacterial genomes is the use of *k*-mers. They provide a comprehensive set of genetic variants in a set of genomes that is not restricted to a single reference genome [7,8]. Further developments have focused on the effective interpretation of significant *k*-mers. These include analysing the genome sequences surrounding *k*-mers through de Bruijn graphs [9,10] and linking each *k*-mer to its gene clusters [11]. While these have improved interpretations of significant *k*-mers, little attempt has been made to interpret *k*-mers in the context of genome rearrangements.

Most genome rearrangements, including inversions and translocations, are thought to occur through within-genome homologous recombination between repeat sequences [12,13], which can often be found at rearrangement boundaries. Frequently encountered repeat sequences that mediate genome rearrangements are insertion sequence elements (IS elements) [14,16], ribosomal RNA operons [17,19], and prophages [20,22]. Recently, microbiologists have turned their attention to genome rearrangements due to their widespread occurrence in bacterial genomes [23,26], their potential for generating phenotypic variation, and hence their evolutionary consequences [20,22, 27]. Apart from disrupting existing genes [28] or creating chimeric genes [29], genome rearrangements can also alter the distance of a gene from the origin of chromosome replication and the DNA strand on which the gene is encoded, which have been shown to influence gene expression [30]. To further understand the phenotypic effects of genome structures, efficient screening for association between genome rearrangements and phenotypic variation at a population level is becoming increasingly desirable.

Recently, there has been growing availability of long-read sequencing technology [31,32] as well as the development of tools that identify and classify bacterial genome structures [19,33]. Hence, testing for association between genome rearrangements' presence–absence patterns and phenotypes could be done in the same way as more commonplace genomic variation-based bGWAS studies (*i.e.* bGWAS based on presence–absence patterns of single nucleotide variants, insertions-deletions, or *k*-mers/unitigs). However, exhaustive and high-throughput genome structure identification and characterization of individual rearrangement events in large datasets remain challenging, meaning that structural variation is not routinely incorporated into bGWAS.

Here, we present a pre- and post-bGWAS processing methodology that makes use of *k*-mers to include genome rearrangements as bGWAS input, without prior definition of genome structure. We use a method that involves replacement of long repeat sequences with short placeholder sequences, so that flanking sequences can be incorporated into *k*-mers. Then, our software maps the location of these flanking regions in *k*-mers identified as significant in bGWAS to complete genome sequences to visualize the rearrangements. We have named the software *GWarrange* and hope that it will be a useful auxiliary bGWAS tool to include genome rearrangements as drivers of phenotypic variation in bacteria.

## Methods

A summary of the pipeline can be found below and in Fig S1. A detailed description of each step, as well as pipeline parameters and output file descriptions, can be found in Supplementary text S1. Detailed guidance can be found in the online tutorials athttps://github.com/DorothyTamYiLing/genome_rearrangement.git https://github.com/DorothyTamYiLing/GWarrange/blob/master/documents/tutorials.md.

Repeat sequence categories in a selected reference genome are identified and used as representatives of repeat sequences in the input genome set. Candidate repeat sequence categories are defined as genetic features that show at least two occurrences in the reference genome based on blast alignment. The identified repeat sequence categories are then used to estimate the number, size, genome locations, and gene members of the repeat sequence clusters in the same reference genome.

The set of input linearized closed genome sequences are re-oriented to start at the same chosen gene, ensuring that it is in the same orientation in every genome sequence. To detect boundaries of genome rearrangements, repeat sequences, which are the most likely sites of recombination, are replaced with short placeholder sequences of Nx15 in each genome sequence. This allows the placeholder sequence itself and its flanking regions to be incorporated into the length of a *k*-mer. Prior to the replacement, the genome coordinates of each repeat sequence are extended by a number of base pairs (bp) in both directions to ensure their complete replacement. Then, neighbouring repeat sequences that are less than a number of base pairs apart are merged into blocks. The number of base pairs for extension is recommended to be similar to and larger than the estimated size of the largest repeat sequence cluster in the selected reference genome (see step above). This results in genome sequences with repeat sequence clusters replaced by placeholder sequences.

Next, *k*-mers are generated from the genome set with repeat sequences replaced using *k*-mer generation tools such as fsm-lite (https://github.com/nvalimak/fsm-lite). The resulting *k*-mers are then used as input for *k*-mer-based bGWAS to search for *k*-mers that are associated with a phenotype of interest. Additionally, in situations that could potentially produce a large number of significant *k*-mers, such as large rearrangement events or the use of large genome sets, we have also incorporated functionality for unitig-based bGWAS to be performed in parallel with *k*-mer-based bGWAS.

Significant *k*-mers are aligned against all the genome sequences in the genome set in their original form (without repeat sequence replacement) using blast. They then undergo different downstream processing depending on whether they contain placeholder sequences or not. When unitig-based bGWAS is performed, unitigs will be analysed instead of, and in the same way as, *k*-mers without placeholder sequences.

**Fig. 1.**
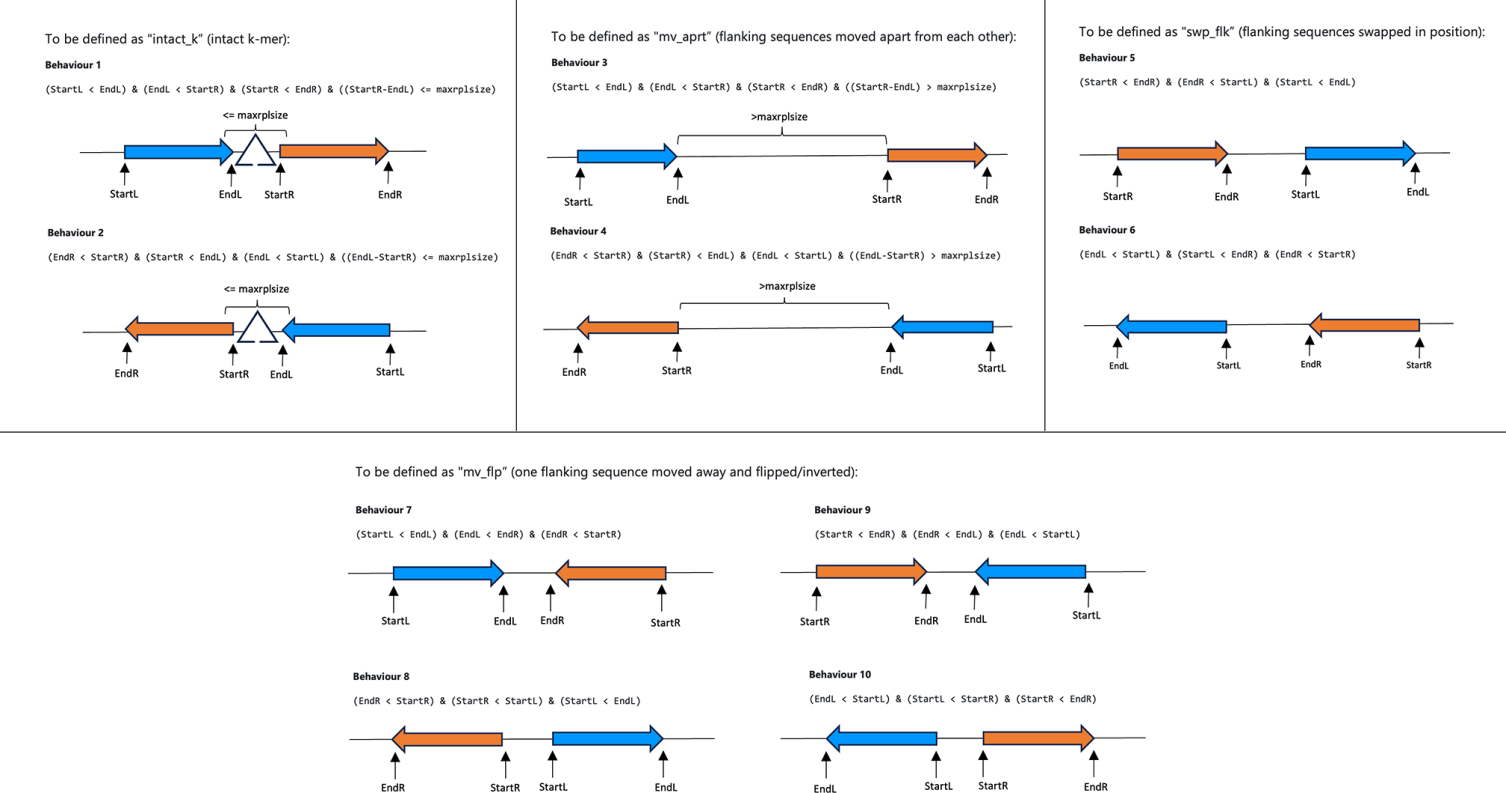
Rules for defining different flanking sequence behaviours, taking into account that *k*-mers might be in forward or reverse orientation when mapped to genome sequences. *k*-mers that show flanking sequence behaviours of ‘mv_aprt’, ‘swp_flk’, or ‘mv_flp’ are defined as split *k*-mers. Blue arrows and orange arrows symbolise left and right flanking sequences, respectively. Note that the difference between ‘intact_k’ and ‘mv_aprt’ is that in ‘intact_k’, the distance between flanking sequences is smaller than or equal to the maximum size of repeat sequence clusters replaced by placeholder sequences (i.e. maxrplsize); whereas in ‘mv_aprt’, the distance between flanking sequences exceeds maxrplsize. It is important to note that behaviours 2, 4, 6, 8, and 10 are mirror behaviours for behaviours 1, 3, 5, 7, and 9, respectively. This is because intact *k*-mers can be in forward or reverse orientation when mapped to genome sequences, hence the same applies to split *k*-mers.

**Fig. 2.**
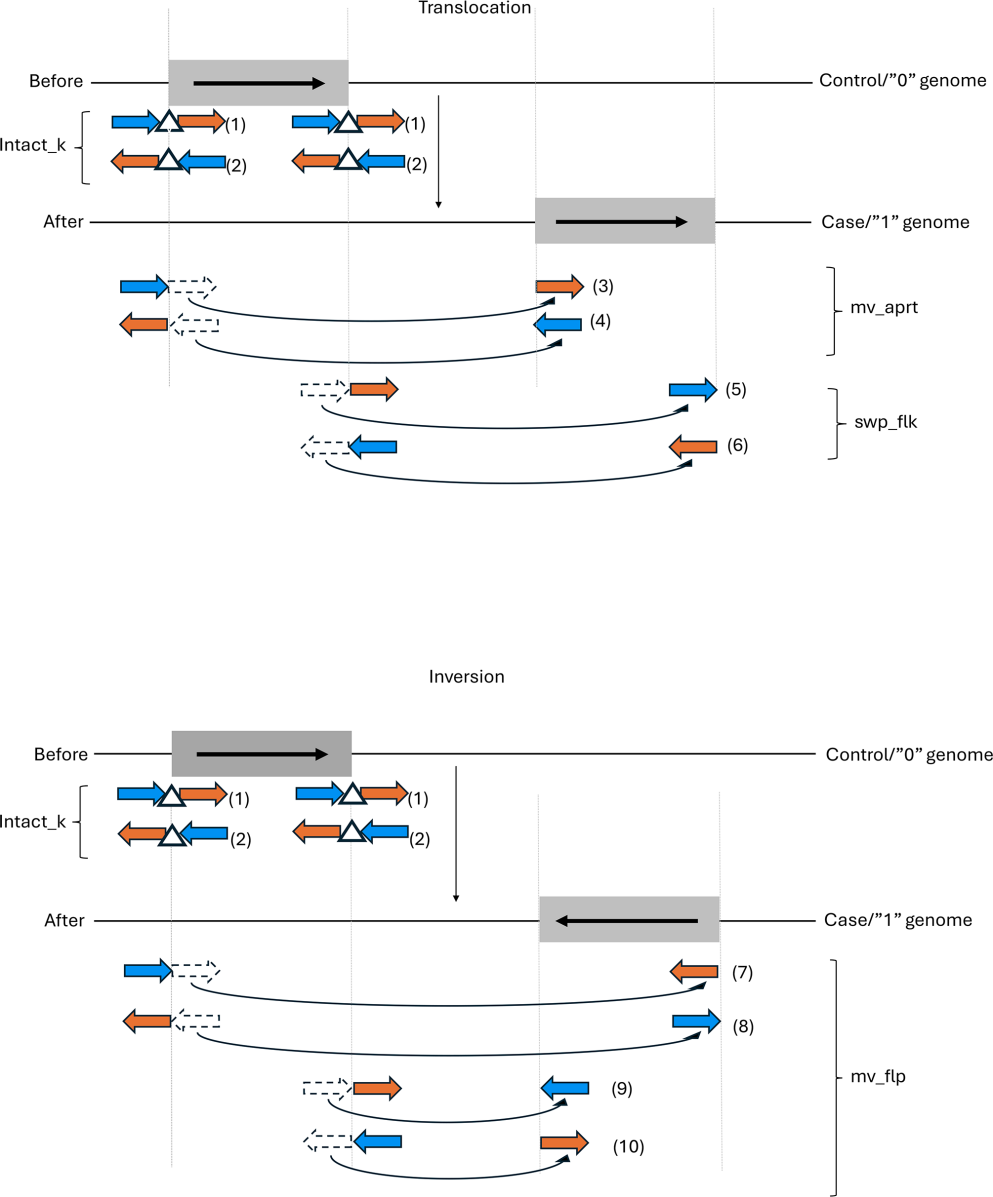
Definition of translocation and inversion by different flanking sequence behaviours when significant *k*-mers containing placeholder sequences are mapped to case and control genomes. Grey boxes symbolize rearranged genome sequences, with the orientation of genome sequences indicated by bold black arrows within the boxes. Blue arrows and orange arrows symbolize left and right flanking sequences, respectively. White triangles symbolize placeholder sequences in intact *k*-mers. Curly black arrows symbolize movements of flanking sequences of a *k*-mer in each behaviour when they are mapped to case and control genomes. Behaviour numbers are in brackets. (Above) When a translocation is observed in case genomes compared to control genomes, *k*-mers containing placeholder sequences that mapped to the rearrangement boundaries in control genomes display ‘intact_k’ behaviour. When these *k*-mers are mapped to case genomes, their flanking sequences either move apart (‘mv_aprt’) or swap positions (‘swp_flk’), suggesting the presence of translocation in case genomes. (Below) When an inversion is observed in case genomes compared to control genomes, *k*-mers containing placeholder sequences that mapped to the rearrangement boundaries in control genomes display ‘intact_k’ behaviour. When these *k*-mers are mapped to case genomes, one flanking sequence moves away and flips/inverts (‘mv_flp’), suggesting the presence of inversion in case genomes. Note that genome rearrangements could also be found in control genomes, hence the reverse of the above will be observed (*i.e.* intact *k*-mers and split *k*-mers observed when *k*-mers are mapped to case and control genomes, respectively).

In summary, for significant *k*-mers containing placeholder sequences, which are indicators of rearrangement boundaries, the flanking sequence behaviour for each *k*-mer in each genome is determined based on its genome coordinate summary and the maximum size of repeat sequence clusters replaced by placeholder sequences (see Fig. 1). Translocation is suggested when a *k*-mer shows ‘intact_k’ behaviour in some genomes and ‘mv_aprt’ or ‘swp_flk’ behaviours in other genomes. In contrast, inversion is suggested when a *k*-mer shows ‘intact_k’ behaviours in some genomes and ‘mv_flp’ behaviour in other genomes (see Fig. 2). *k*-mers that show flanking sequence behaviours of ‘mv_aprt’, ‘swp_flk’, or ‘mv_flp’ are defined as split *k*-mers. For each of these split *k*-mers, the count and proportion of case and control genomes showing each flanking sequence behaviour and summary statistics of their genome positions are generated. For significant *k*-mers containing placeholder sequences that show ‘intact_k’ behaviour in all genomes, they are analysed the same way as those without placeholder sequence. For *k*-mers/unitigs that do not contain placeholder sequences, which are indicators of rearranged sequence content, a summary is generated for the count and proportion of case and control genomes containing the *k*-mers in forward or reverse orientation, as well as summary statistics of their genome positions.

The identified genome rearrangement events are finally visualized. For split *k*-mers containing placeholder sequences, their visualization involves plotting where the flanking sequences are found in case and control genomes and their orientations. To simplify the output, these *k*-mers are deduplicated so that each rearrangement boundary is represented by the plots of one to two *k*-mers in forward and/or reverse orientation. For intact *k*-mers, both with and without a placeholder sequence, as well as unitigs, their visualization entails plotting where the *k*-mers are found in case and control genomes and their orientations after deduplication.

## Results

The pipeline has been applied to three datasets consisting for two bacterial species: *B. pertussis* and *E. faecium*, demonstrating the detection of phenotype-associated inversions. Structural variation has been observed in both species, and their genomes are enriched with IS elements [34,35]. In the fourth example, the pipeline was applied to a simulated dataset to demonstrate the behaviours of flanking sequences describing translocations. In the first two examples, genome structure was used as the phenotype input for GWAS.

*k*-mers of 200 bases were generated using fsm-lite (https://github.com/nvalimak/fsm-lite), with a minor allele frequency of 0.05 applied. Then, a *k*-mer-based GWAS was conducted using pyseer [36] to identify *k*-mers whose presence–absence patterns showed association with the phenotype of interest or genome structure phenotype, which are either ‘0’ or ‘1’ as defined in the input phenotype file. Unitigs generated by unitig-caller [37] were used in combination with k-mers in example 2. A minimum merge parameter of 3 bp was used to merge adjacent and overlapping repeat sequences. Association statistics for each significant *k*-mer/unitig can be found in sigk_pyseer.txt/siguni_pyseer.txt in * ISreplaced_genomes* directory.

To use different tools for generating *k*-mers/unitigs or for performing GWAS, users can run each step separately by following the instructions in the ‘tutorial’ section on the GitHub page.

Descriptions for all textual output files, including interpretations for each column, can be found in the ‘Pipeline and output file description’ section on the GitHub page.

**Example1:***B. pertussis* (predefined genome structure as phenotype)

(Run time with 8 CPUs : 10 min)

The pipeline was applied to a dataset of closed *B. pertussis* genome sequences as described in [35], where genome structures were defined by MAUVE exhaustive pairwise alignment [38]. A subset of 47 genome sequences was used, displaying two different genome structures: 29 genome sequences with structure ‘0’ and 18 genome sequences with structure ‘1’, explained by two nested inversions (see Fig. S3). In this case, genome structure was used as the phenotype input for GWAS. To simulate real-world scenarios, the assigned genome structure of two pairs of genome sequences was swapped to add artificial noise to the dataset, aiming to better represent most experimental datasets (see Table S1).

Complete genome assemblies were re-oriented to begin with the first base of the initiating codon of the gene *gidA,* adjacent to the origin of replication. In the reference genome C505 (accession: NZ_CP011687.1), the IS481 family transposase, IS481-like element IS481 family transposase, and IS110-like element IS1663 family transposase were identified as the most ubiquitous repeat sequence categories. The size of the largest repeat sequence cluster was 5735 bp. Two different extension parameters of 100 bp (minimum extension for preserving breakpoints) and 7000 bp (for ensuring complete masking of repeat sequence blocks) were used, capturing slightly different information (see below). Population structure was not controlled because the structural variation observed in this dataset was tied to the population structure.

### Split *k*-mers visualizing rearrangement boundaries

When using a 7000 bp extension, 976 significant *k*-mers containing placeholder sequences were found to be split and showed ‘mv_flp’ behaviour in at least one genome (*i.e. k*-mers with flanking sequences mapped to different positions and in different orientations in the genomes). By plotting deduplicated split *k*-mers (see Supplementary text S1 for deduplicated split *k*-mers definition), four inversion boundaries were found that potentially referred to two inversion events, *i.e.* between genome coordinates 430 and 3600 Kbp, as well as between 1500 and 2500 Kbp, with one inversion nested within the other. These boundaries matched the known inversions depicted in Fig. S3. The four boundaries could be indicated by 16 different significant split *k*-mers that were mapped to each of the boundaries, split in case/control genomes, and in forward/reverse orientation. Plots of four example split *k*-mers are shown in Fig. S4 (output files in /kmers_withN/splitk_plots directory). Plots for all sixteen split *k*-mers can be found in Fig. S5.

Alternatively, when using a 100 bp extension, only two inversion boundaries that referred to an inversion between 430 and 3600 Kbp were detected by significant split *k*-mers containing placeholder sequences. Through manual sequence investigation of the 1500 and 2500 Kbp boundary, it was found that the boundaries contained additional repeat sequences (*i.e.* FUSC family membrane protein, MFS transporter, peroxide stress protein YaaA) located immediately adjacent to IS elements (Fig. S6). These additional repeat sequences were several thousand base pairs in size, hence minimal extension and merging were not sufficient to completely mask them, resulting in them being present in flanking sequences. Since effective detection of genome rearrangement boundaries relies on unique mapping of flanking sequences to genomes, these inversion boundaries at 1500 and 2500 Kbp can only be detected when the whole repeat sequence blocks are completely replaced (*i.e.* by using a 7000 bp extension).

### Intact *k*-mers (with and without placeholder sequence) visualizing rearranged sequence content

When using a 100 bp extension, 4166 intact *k*-mers were output as significantly associated with the genome structure ‘phenotype’, while when using a 7000 bp extension, 1206 intact *k*-mers were returned. The higher number of significant intact *k*-mers found using a 100 bp extension was due to the 7000 bp extension resulting in a lower overall number of *k*-mers being produced. Results of mapping deduplicated intact *k*-mers (with and without placeholder sequence combined) to genomes are shown in Fig. S7 (output files in /allintack_combinedplots directory).

**Fig. 3.**
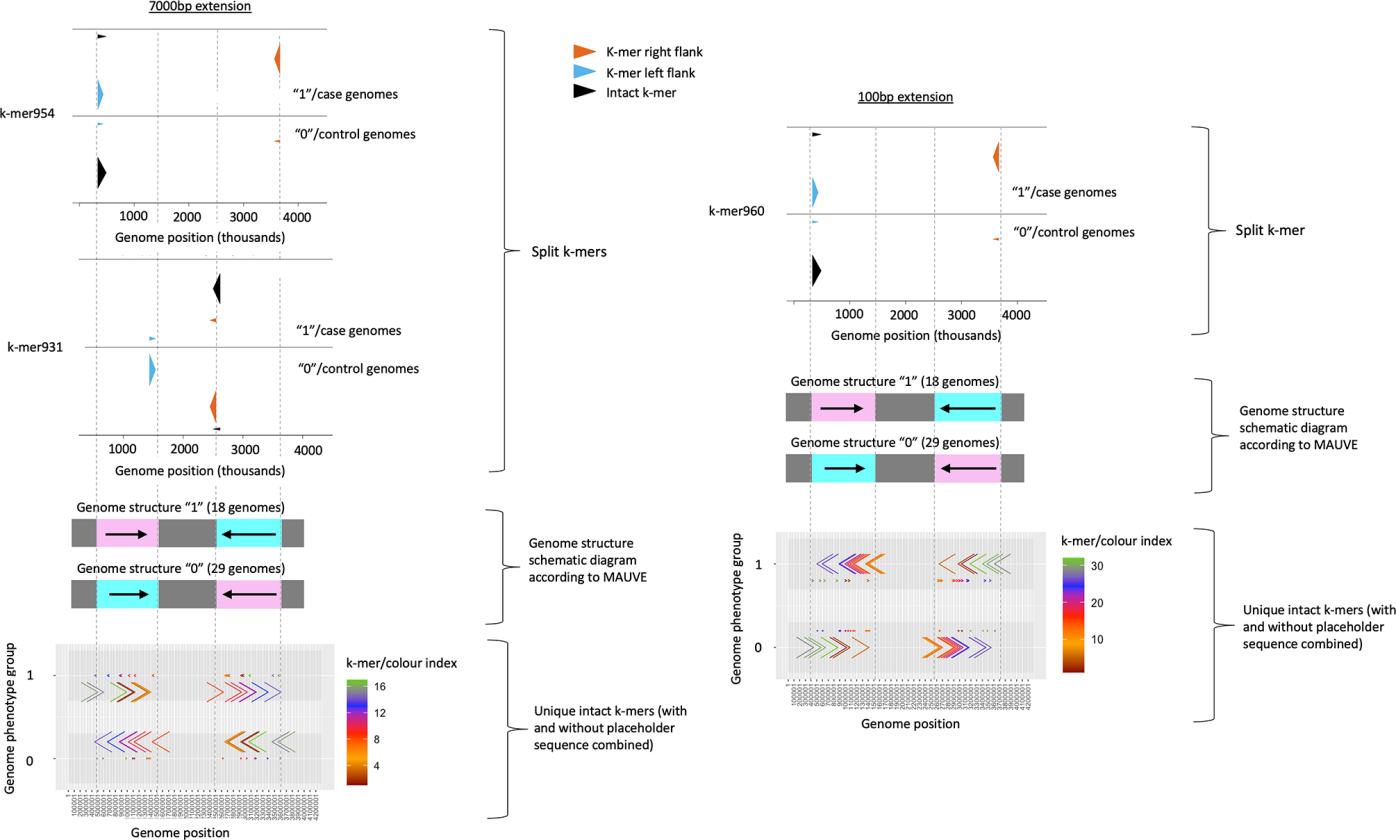
Rearrangement boundaries and rearranged sequence content that were captured to different extents by using different extension parameters. When using a 7000 bp extension, all the rearrangement boundaries at genome positions 430, 3600, 1500, and 2500 Kbp were captured; when using a 100 bp extension, only the rearrangement boundaries at genome positions 430 and 3600 Kbp were captured. The genome phenotype groups ‘1’ and ‘0’ refer to case and control genomes, respectively.

Information from split and intact *k*-mers could be combined to visualize rearrangement boundaries and rearranged sequence content, which were captured to varying extents by using different extension and merging parameters (see Fig. 3).

**Example 2:***E. faecium* (predefined genome structure as phenotype)

(Run time with 8 CPUs : 15 min)

The genome structures of 75 *E. faecium* strains were previously characterized using a combination of long-read sequencing and the software Socru, designed for typing the order and orientation of genome fragments between ribosomal operons [19]. Through manual sequence checking, IS elements were also found at some of the rearrangement boundaries. Similar to example 1, the genome structure genotype was used as the phenotype to test whether the pipeline is capable of detecting characterized genome rearrangements. A subset of 32 genomes displaying two different chromosome structures (21 genomes with structure ‘0’ and 11 genomes with structure ‘1’) were used in this example (see Fig. S8). The genome structure phenotype of two pairs of genomes was swapped to add artificial noise to the dataset that would normally be observed in real-life GWAS datasets (see Table S2).

Complete genome assemblies were re-oriented to begin with the coding sequence of *dnaA*. In the reference genome AUSMDU00004142 (accession: NZ_CP027501.1), ISL3-like element ISEfa11 family transposase, IS256-like element ISEf1 family transposase, and IS30 family transposase were identified as the most ubiquitous repeat sequence categories, with the size of the largest repeat sequence cluster being 15 773 bp. Two different extension parameters of 100 bp (minimum extension for preserving breakpoints) and 17 000 bp (for ensuring complete masking of repeat sequence blocks) were used, although it was shown that the minimum extension was sufficient to capture genome rearrangements (see below). Population structure was not controlled because the structural variation observed in this dataset was tied to the population structure.

A substantial number of significant *k*-mers were obtained when using a 100 bp extension, but few contained a placeholder sequence (424 588 significant *k*-mers, 1243 with a placeholder sequence). Analysing such a large number of *k*-mers would necessitate a lengthy runtime (approximately 60 000 to 70 000 *k*-mers processed per hour with 8 CPUs for this genome set). The pipeline was subsequently rerun using unitig-based GWAS. Instead of analysing significant *k*-mers without placeholder sequences, unitigs identified as significant were analysed alongside significant *k*-mers containing placeholder sequences, which enable the visualization of rearrangement boundaries.

### Split *k*-mers visualizing rearrangement boundaries

Using a 100 bp extension, 215 significant *k*-mers containing placeholder sequences were found to be split and exhibited ‘mv_flp’ behaviour in at least one genome (*i.e*. the flanking sequences of the *k*-mer mapped to different positions and orientations in the genomes). By plotting four deduplicated split *k*-mers, two rearrangement boundaries were identified, indicating a single inversion event between genome coordinates 720 and 2100 Kbp, consistent with the known inversion depicted in Fig. S8. All split *k*-mers were observed to be split in case genomes and intact in control genomes. This could be attributed to the absence of IS elements at rearrangement boundaries in case genomes, as confirmed by manual inspection of the genome sequences. The two boundaries can be indicated by four different significant split *k*-mers that were mapped to each of the boundaries in both forward and reverse orientations (see Fig. S9) (output files in /kmers_withN/splitk_plots directory). No significant split unitig was identified as unitig-based *GWarrange* is only applicable to genome sequences without placeholder sequences (*i.e.* unitig-caller is not able to generate unitigs containing placeholder sequences).

### Unitigs for visualizing rearranged sequence content

Using a 100 bp extension, 3737 significant unitigs were returned, and deduplicated unitigs were plotted to visualize rearranged sequence content. A much smaller number of significant unitigs were found using the larger extension parameter (*i.e.* 17 000 bp) as fewer unitigs were produced initially (see Fig. S10) (output files in /kmers_noN directory).

**Fig. 4.**
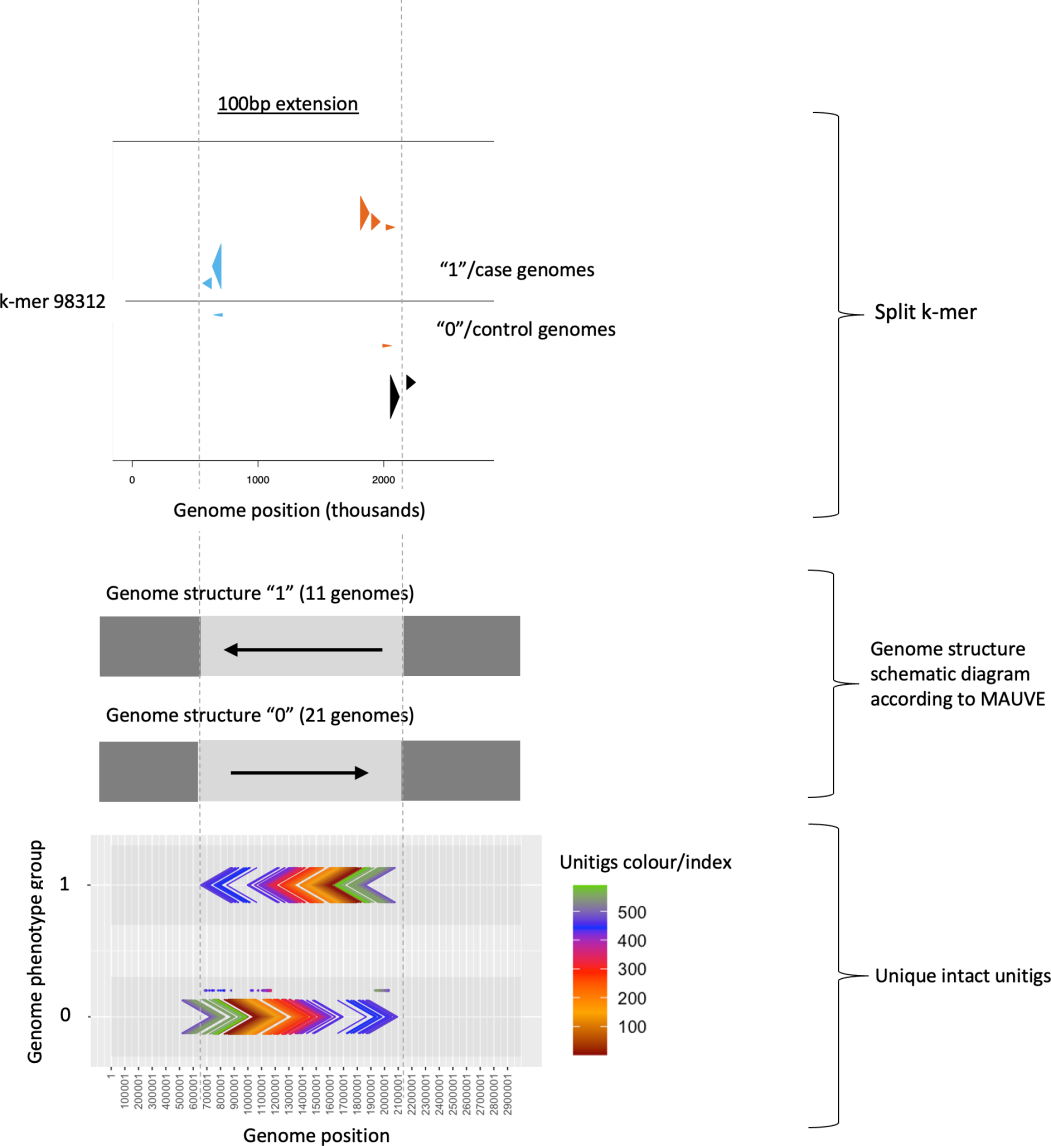
Combining information from significant split *k*-mers (one split *k*-mer example is shown) and unitigs from the pipeline using a 100 bp extension. Split *k*-mers captured rearrangement boundaries at genome positions 720 and 2100 Kbp, while rearranged sequence content is indicated by unitigs. Genome phenotype groups ‘1’ and ‘0’ refer to case and control genomes, respectively.

By combining rearrangement information obtained from significant split *k*-mers and unitigs, both rearrangement boundaries and rearranged sequence content can be captured (see Fig. 4).

**Example 3:***B. pertussis* (pertactin expression phenotype)

(Run time with 8 CPUs : 120 min)

Pertactin, encoded by the pertactin (*prn*) gene, is a *B. pertussis* outer membrane protein and one of the antigens in the acellular pertussis vaccines. Over the past 20 years, the proportion of *B. pertussis* isolates deficient in the expression of pertactin has dramatically increased in numerous countries due to a number of different mutations, such that in some, a large majority of recent isolates are Pertactin deficient. This has been attributed to acellular vaccine-mediated selection pressure [39]. The pipeline was applied to a dataset of 468 *B. pertussis* closed genome sequences using pertactin expression status as described in [40] as the phenotype. Among them, 165 strains expressed pertactin, while 303 were deficient in expression.

The same steps were conducted as for example 1, followed by bGWAS with population structure control applied using a phylogenetic similarity matrix. Truncation information of the *prn* gene from NCBI annotation was used as a covariate (233 genomes with *prn* truncation, 235 genomes without) (Table S3). The pipeline was run twice using the two different sets of merging and extension parameters as used in example 1.

**Fig. 5.**
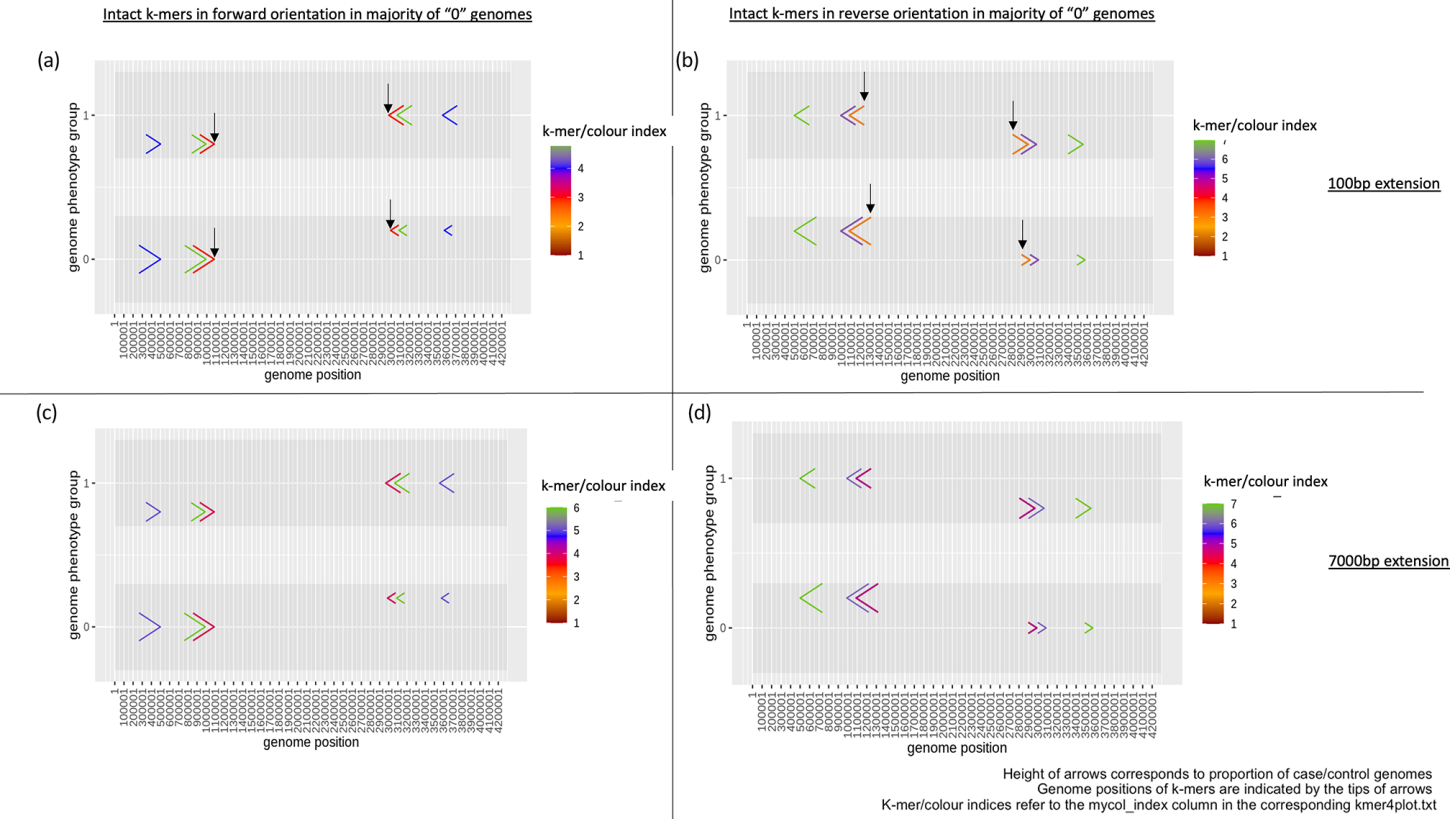
Plots of intact deduplicated *k*-mers without placeholder sequence that showed rearranged sequence content significantly associated with pertactin expression phenotype, when using 100 bp (**a and b**) and 7000 bp (**c and d**) extension, and when intact *k*-mers are in forward (**a and c**) and reverse orientation (**b and d**) in the majority of control genomes (genome phenotype group ‘0’). The direction of coloured arrows indicates the orientation of *k*-mers when mapped to genomes. In case genomes (genome phenotype group ‘1’) in all plots, half of the genomes have significant *k*-mers aligned in the forward orientation, while the other half are in reverse orientation at the opposite end of the genome; whereas for control genomes in all plots, a larger difference in the proportion of case and control genomes with *k*-mers that aligned in different directions and genome positions was observed. *k*-mers containing pertactin autotransporter gene sequences (indicated by vertical black arrows) were captured only from the genome set with a 100 bp extension (**a and b**) because the gene was completely embedded within the region for replacement when a large extension parameter of 7000 bp (**c and d**) was applied, thus any change in the position of the gene due to genome rearrangement could not be detected (Fig. S11).

When using a 100 bp extension, no significant split *k*-mer was returned, but 2526 intact *k*-mers without placeholder sequence were found to be significantly associated with the phenotype (*p*-value threshold for significance=1.32E-04). Twelve deduplicated intact *k*-mers were plotted to visualize rearranged sequence content (Fig. 5) (output files in /kmers_noN directory). Although no rearrangement boundary was found, it was speculated that an inversion event might have taken place that affected the position and orientation of genome sequences indicated by the mapped intact *k*-mers: while significant intact *k*-mers showed different orientations and genome positions in half of the case genomes, they were seen in the same orientation and genome position in the majority of control genomes (Fig. 5). A number of these *k*-mers were found to contain pertactin autotransporter gene sequences. However, these *k*-mers were not found when using a 7000 bp extension, as the gene was located immediately next to a repeat sequence in the *B. pertussis* genomes. When a large extension parameter was used, *e.g.* 7000 bp, the gene was completely embedded within the region for replacement, such that any change in position of the gene due to genome rearrangement could not be detected. In this case, the larger merging threshold limited the detection of rearranged sequence content (Fig. S11).

**Example 4:**

Simulated genome set displaying translocation (presence and absence of simulated translocation as phenotype)

(Run time with 8 CPUs: 20 min)

In this example, 39 simulated genomes were utilized to illustrate the flanking sequence behaviours of *k*-mers during translocation. Simulated genomes were used because the genomes susceptible to translocation typically undergo significant perturbation due to the high frequency of translocations, as observed in the genomes of *Salmonella enteric*a [19]. To detect phenotype-associated translocations in *k*-mer-based GWAS, the presence–absence pattern of *k*-mers at the rearrangement boundary should mirror that of the phenotype-associated translocation. For instance, a *k*-mer at the translocation boundary remains intact or present (*i.e.* intact_k) in genomes before translocation and becomes split (*i.e.* ‘mv_aprt’ or ‘swp_flk’), and consequently absent in genomes after translocation. In a scenario with a high occurrence of translocations, the presence–absence status of the *k*-mer is likely to be influenced by background rearrangement events, thus resulting in reduced sensitivity in detecting phenotype-associated translocations.

Genome structures ‘0’ and ‘1’ were represented by 19 and 20 genomes, respectively, with the discrepancy attributed to two translocation events (Fig. S12). Simulated genome sequences were obtained from *Bordetella pertussis* genomes, while ribosomal operons, mainly consisting of 16S ribosomal RNA, 23S ribosomal RNA, 5S ribosomal RNA, and tRNAs, were sourced from *Salmonella enterica* genomes and inserted into each of the rearrangement boundaries resulting from translocation. In the reference simulated genome sim1.fasta, IS200/IS605 family transposase, 16S ribosomal RNA, 23S ribosomal RNA, and 5S ribosomal RNA were identified as the most prevalent repeat sequence categories. The largest repeat sequence cluster was 5494 bp in size. Two different extension parameters, 100 bp (to preserve breakpoints) and 7000 bp (to ensure complete masking of repeat sequence blocks), were employed. Population structure was not controlled since the structural variation observed in this dataset was associated with the population structure. Simulated genome structure served as the phenotype to directly evaluate whether the pipeline could detect predefined genome rearrangements. The structural phenotype of two pairs of genomes was swapped to introduce artificial noise into the dataset, akin to what would typically be observed in a real-life GWAS dataset (Table S4).

**Fig. 6.**
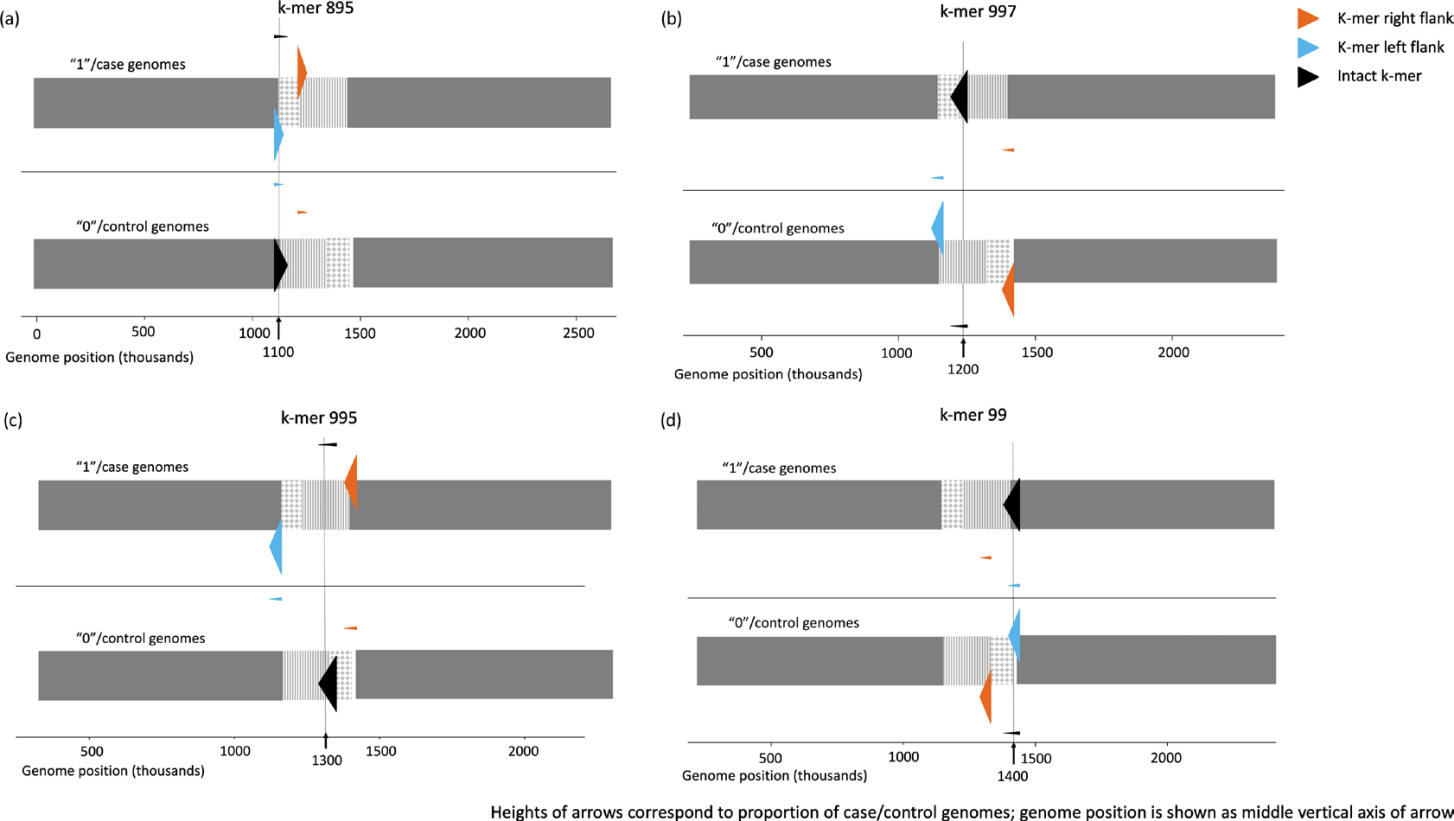
Example plots of four split *k*-mers indicating four translocation boundaries, from the genome set using a 7000 bp extension. (a) At the 1100 K boundary, the "mv_aprt" *k*-mer remains intact in the majority of control genomes in the forward orientation, while it is split in the majority of case genomes. (b) At the 1200 K boundary, the "swp_flk" *k*-mer remains intact in the majority of case genomes in reverse orientation and is split in the majority of control genomes. (c) At the 1300 K boundary, the "swp_flk" *k*-mer remains intact in the majority of control genomes in reverse orientation and is split in the majority of case genomes. (d) At the 1400 K boundary, the "mv_aprt" *k*-mer remains intact in the majority of case genomes in reverse orientation and is split in the majority of control genomes.

Using a 7000 bp extension, 12 split *k*-mers were identified, displaying ‘mv_aprt’ and ‘swp_flk’ behaviour in at least one genome. By mapping deduplicated split *k*-mers to simulated genomes without replacement, four rearrangement boundaries, corresponding to those observed in Fig. S12, were identified. Four split *k*-mer examples indicating the boundaries were shown in Fig. 6 (output files in /kmers_withN/splitk_plots directory). Plots for all 12 split *k*-mers are shown in Fig. S13.

No significant intact *k*-mers without placeholder sequence were found. This outcome was expected since the current method is unsuitable for detecting rearranged sequence content in translocations that do not involve any change in sequence orientation.

## Discussion and conclusion

Our results demonstrate that, with complete closed genomes, our *k*-mer-based pre- and post-GWAS processing pipeline can detect phenotype-associated rearrangements mediated by a list of candidate repeat sequence categories identified from a selected reference genome. We have demonstrated this by using genome structure as the phenotype in a series of genome rearrangement examples, real-life GWAS datasets, as well as using simulated genomes. The detection encompasses both rearrangement boundaries and rearranged sequence content.

Detection of rearrangement boundaries requires the repeat sequences/repeat sequence blocks that have mediated the rearrangements to have been completely replaced by placeholder sequences. Therefore, the implementation of repeat sequence extension and merging can enhance detection sensitivity, as illustrated in example 1. We have demonstrated the use of the maximum repeat cluster size in a reference genome to estimate the extension value for optimal rearrangement detection results. However, it is advisable to exercise caution when performing repeat sequence replacement, as large-scale extension and merging may result in the loss of precise rearrangement breakpoint locations. Additionally, any genome rearrangement entirely contained within the replaced region will go undetected, as demonstrated in the *B. pertussis* pertactin example (example 3). By default, minimal extension and merging parameters are also applied to preserve rearrangement breakpoints to the fullest extent possible.

This method can also incorporate the use of unitigs, which makes it scalable to datasets with a large number of genomes, genomes of large size, and/or genomes containing large rearrangement events, as demonstrated by the *E. faecium* example (example 2). Nevertheless, visualization of rearrangement boundaries relies on *k*-mers, as unitigs are not designed to incorporate placeholder sequences. This pipeline is proven effective in detecting phenotype-associated inversions; however, the detection of translocations might be challenging as genomes that are prone to translocations are usually highly perturbed by a high frequency of translocation that might or might not show an association with the phenotype of interest. This could add noise to the presence–absence status of *k*-mers, hence reducing sensitivity in detecting phenotype-associated translocation boundaries. This method is also not suitable for detecting rearranged sequence content in translocations as they do not involve any change in sequence orientation, which can be captured by *k*-mers/unitigs counting tools and translated into their presence/absence status (example 4). It should be noted that false positives can occur if whole genomes are not reoriented so that they all begin with the same gene. Lastly, despite population structure not being controlled for in examples 1, 2, and 4, they are exceptional scenarios in which genome structures were used as phenotype input for GWAS; hence, correcting for population structure could remove causal associations. In practice, all bGWAS should incorporate population structure correction to control for confounding associations.

In summary, this novel method will offer insights into understanding how genome rearrangements contribute to phenotype, providing valuable information about bacterial populations and evolutionary dynamics.

## supplementary material

10.1099/mgen.0.001268 Fig. S1.

10.1099/mgen.0.001268 Uncited Table S1.

## Abbreviations

bGWAS: bacterial genome wide association studies
BLAST: Basic Local Alignment Search Tool
IS elements: insertion sequence elements
