# Supplementary material for: GWarrange: a pre- and post- genome-wide association studies pipeline for detecting phenotype-associated genome rearrangement events
Source: Microb Genom. 2024 Jul 9;10(7):001268. doi: 10.1099/mgen.0.001268 (PMC11316554; doi:10.1099/mgen.0.001268)
Supplement: Fig. S1. [file mgen-10-01268-s001.pdf]

Supplementary text S1: pipeline description

1. **Identify candidate repeat sequence categories in selected reference genome**

Sequences for all features within the chosen reference genome are extracted from its complete genome assembly based on coordinates provided in a .gff file. They are then placed in a multiFASTA file (query) prior to aligning with the assembly (subject) through BLAST in order to search for sequences with repeat occurrences. Sequences that show at least two alignments with an identity and query coverage of at least 80% are retained. The list of sequences is then subjected to deduplication through pairwise BLAST alignment among themselves and by the following procedure. Initially, one sequence is randomly selected and labelled as "unique". Any other sequences within the list showing greater than 80% identity and query coverage with this selected sequence are marked as "duplicate". Following this, the "unique" and "duplicate" sequences are removed from the list, and the process is iterated until all sequences are categorised as either "unique" or "duplicate". The final set of “unique” sequences are the representatives of repeat sequence categories in the reference genome. Output files include homo_occurrence.txt in the /output_homo directory, which lists the identified deduplicated repeat sequence categories, and homodedup.fna within the same directory, containing the repeat sequences in FASTA format. The default values for identity and query coverage are set at 80%, although these can be modified using the -idcov flag.

2. **Estimating the size of repeat sequence clusters in the reference genome**

The representatives of identified repeat sequence categories undergo realignment with the chosen reference genome through BLAST, maintaining criteria of 80% identity and query coverage. Alignments that are less than 1000 base pairs (bp) apart in the reference genome are defined as belonging to a single repeat sequence cluster. Details such as the number, size and genome locations of these clusters are recorded in the output file homo_cluster.txt within the /output_homo directory. The default values for identity and query coverage are set at 80% (can be adjusted using the -idcov flag), while the default distance threshold of 1000 bp can be modified using the -dist flag.

3. **Reorientation of input genome assemblies**

To identify genome rearrangements, a set of closed genome sequences is required, as draft, multi-contig genome sequences do not contain the structural information to the degree required for thorough recognition of rearrangements. Since most bacterial closed genome sequences are depicted as linearised genomes, they are re-orientated to start at the same chosen gene, ensuring the same orientation in every genome sequence. The gene of choice is largely flexible and should have minimal impact on the reproducibility of bGWAS, provided that it is present in a single copy, highly conserved, and stable across genomes, such as *dnaA* in *Enterococcus faecium* or *gidA* in *Bordetella pertussis*. The location and orientation of the chosen gene are obtained by aligning the gene sequence to the genome assemblies through BLAST (using default settings, since only one alignment per genome should be expected).

Then, assemblies are re-orientated according to the location and orientation of the chosen gene in the BLAST output.

4. **Replacement of repeat sequences by short placeholder sequences across the genome set**

To detect boundaries of genome rearrangements, repeat sequences likely to be recombination sites are replaced with short placeholder sequences of N x 15 in each genome sequence. This allows for the placeholder sequence itself and its flanking regions to be incorporated into the length of a k-mer. Locations of repeat sequences across the genome set are obtained through BLAST using default settings. The more relaxed percentage identity and coverage thresholds here allow the detection of fragmented repeat sequences in the genome. The list of repeat sequences used in BLAST is supplied by the user in FASTA format (using the -replist flag) and can be informed by the most frequent repeat sequence categories identified in the selected reference genome in the above step.

5. **Extending and merging neighbouring repeat sequences into blocks prior replacement**

Repeat sequences, such as IS elements, can sometimes be found in clusters in bacterial genomes, or different types of repeat sequences can co-locate next to one another forming repeat sequence blocks. Since effective detection of genome rearrangement boundaries relies on regions flanking repeats mapping uniquely to query genomes, it is necessary to replace the whole repeat sequence block/cluster with placeholder sequences. This can ensure: 1) flanking regions can be incorporated into the length of a k-mer; 2) unique mapping of flanking regions to genome sequences due to the absence of repeat sequences. Complete replacement of repeat sequence blocks can be achieved by extending genome coordinates of each repeat sequence in each genome by a number of base pairs in both directions, and/or merging repeat sequences that are less than a number of base pairs apart. Different extension and merging parameters can be specified by users. The number of base pairs for extension is recommended to be similar to or larger than the estimated size of the largest repeat sequence clusters in the selected reference genome (see step above). Unless otherwise specified, repeat sequences that are less than 3bp apart (default) are merged. Note that this also merges overlapping repeat sequences. This results in genome sequences with repeat sequence clusters replaced by placeholder sequences.

Although extending repeat sequences and merging repeat sequences in close proximity can improve sensitivity in rearrangement boundaries detection, too large an extension in both directions and/or merging repeat sequences that are too far apart can lead to loss of resolution of exact rearrangement breakpoints. In addition, any rearrangement that sits completely within the replaced region could not be detected. By default, a minimal extension of 100bp in both directions is performed**,** as well as merging overlapping/adjacent

repeat sequences of 3bp or less apart is also performed. They produce a second independent set of genome sequences containing placeholder sequences.

The coordinates of each extended and merged repeat sequence block (in *mergedIS.txt file), as well as their maximum and minimum size, and distances between them across all genomes (in *mergedISstat.txt file) are recorded.

6. K-mer generation and bGWAS

Next, k-mers are generated from the genome set with repeat sequences replaced using k-mer generation tools such as fsm-lite (https://github.com/nvalimak/fsm-lite). The chosen size of k-mer should allow it to contain the placeholder sequence (if there is any), as well as the two flanking sequences. The size of flanking sequences should be long enough to generate confident sequence match with the genome sequences during BLAST alignment, but short enough that they do not contain a second placeholder sequence, or any remaining repeat sequence that could result in multiple sequence matches. For example, for *B. pertussis* and *E. faecium*, a k-mer size of 200bp (*i.e.* default value for -fsmlite_arg flag) and minimum flanking sequence of 30bp (*i.e.* default value for -flk_len flag) can be used. The resulting k-mers are then used as input for k-mer-based bGWAS to search for k-mers that are associated with a phenotype of interest. In addition, in situations that could potentially produce a large number of significant k-mers, such as large rearrangement events or the use of large genome sets, we have also incorporated functionality for unitigs-based bGWAS to be performed in parallel with k-mers-based bGWAS (See Example 2 in the Result section). Instead of uncompacted k-mers, the use of unitigs could increase efficiency in visualisation of rearranged sequence content (*i.e.* translocated or inverted sequences), as well as reduce computational time. Unitigs are generated from genome assemblies using tools such as unitig-caller (37). To our knowledge, unitig generation tools such as unitig-caller (37) are not compatible with genome sequences containing placeholder sequences. Therefore, unitigs have to be used in combination with k-mers, which are compatible with genome sequences containing placeholder sequences and allow visualisation of rearrangement boundaries.

Significant k-mers are placed into different multiFASTA files according to whether they contain placeholder sequences or not. The two files then undergo different downstream processing. When unitig-based bGWAS is performed, unitigs will be analysed in the same way as k-mers without placeholder sequences.

7. Processing significant k-mers containing placeholder sequences (Indicators of rearrangement boundaries)

k-mers that contain placeholder sequences are retained only if they have flanking sequences of a minimum number of base pairs in size on both sides of the placeholder sequence, as indicated by the -flk_len flag. For example, for *B. pertussis* and *E. faecium*, 30bp (default) can be used as input for the -flk_len flag. This is to ensure that the flanking sequences are long enough to generate confident, unique blast hits. These k-mers are then blasted against all

the genome sequences in the genome set in their original form (without repeat sequence replacement) using default BLAST parameters.

The k-mers are further filtered based on BLAST alignment information using 4 criteria:

- the k-mer should produce BLAST alignment with ≥ 95% of the genomes in the dataset (when a k-mer does not show any BLAST alignment with a genome, it is likely to indicate an insertion-deletion event and is not informative of genome rearrangements. Therefore, it is equivalent to missing information for the genome. Missing information in no more than 5% of the genomes is allowed for a k-mer to pass the filter)
- the k-mer should produce two BLAST alignments in each genome, one for each flanking sequence
- each BLAST alignment should be ≥ 90% of the flanking sequence's length
- each BLAST alignment should show ≥ 95% identity match with an E-value of ≤ 10e-10

For each k-mer that has passed the filter, a summary derived from its blast alignment to each genome is made for 1) StartL (genome coordinate of the start of left flanking sequence) 2) EndL (genome coordinate of the end of left flanking sequence) 3) StartR (genome coordinate of the start of right flanking sequence) 4) EndR (genome coordinate of the end of right flanking sequence) (Figure S2).

Based on the genome coordinate summary of each k-mer and the maximum size of repeat sequence cluster replaced by placeholder sequences (*i.e.* maxrplsize, can be found in *mergedISstat.txt file), the flanking sequence behaviour for each k-mer in each genome is determined. Behaviours could be "intact_k" (intact k-mer), "mv_aprt" (flanking sequences moved apart), "swp_flk" (flanking sequences swapped in position) and "mv_flp" (one flanking sequence moved away and flipped/inverted). Behaviour for each k-mer in each genome is defined according to the different orders of StartL, EndL, StartR and EndR genome coordinates, as shown and visualised in Figure 1. K-mers that show flanking sequence behaviours of "mv_aprt", "swp_flk" or "mv_flp" are defined as split k-mers. When a k-mer shows "intact_k" behaviour in some genomes and "mv_aprt" or "swp_flk" behaviours in other genomes, translocation is suggested by the k-mer; whereas when a k-mer shows "intact_k" behaviours in some genomes and "mv_flp" behaviour in other genomes, inversion is suggested by the k-mer (Figure 2). Flanking sequence behaviours are defined as "undefined_behave" when none of the rules are fulfilled for that k-mer. This should be unlikely, but if it does happen, BLAST results including StartL, EndL, StartR and EndR for that k-mer in each genome will be generated for investigation (output file name: myundefine_k.txt in /kmers_withN directory).

For each k-mer that is defined as either "intact_k", "mv_aprt", "swp_flk" or "mv_flp", the number and proportion of case and control genomes displaying that behaviour are calculated. The most parsimonious genome rearrangement event that is associated with the

phenotype is recorded (*i.e.* “mv_aprt” or “swp_flk” represents translocations, whereas “mv_flp” represents inversions, Figure 2). In addition, the information of where the flanking sequence behaviours occur in the genomes (in the form of summary statistics of genome coordinates) is also summarised.

If a behaviour is found in fewer than 20% of both case and control genomes, it is not included in the summary for the k-mer, as it is unlikely to be associated with the phenotype of interest.

Summaries for significant k-mers with placeholder sequences showing split k-mers behaviours in at least 20% of case or control genomes can be found in output file mysplitk_out.txt in /kmers_withN directory. Descriptions for each column can be found in the "Pipeline and output file description" section on GitHub.

For significant k-mers containing placeholder sequences that show “intact_k” behaviour in all genomes, they are analysed the same way as those without placeholder sequence (see below). Summaries of count and proportion of case and control genomes containing the k-mers in forward/reverse orientation, as well as summary statistics of their genome positions are stored in the output file myintactkwithN_out.txt in / kmers_withN directory.

8. **Processing k-mers/unitigs that do not contain placeholder sequences (Indicators of rearranged sequence content)**

It has been observed that for k-mer or unitig (denoted as k-mers in this section) generation tools such as fsm-lite (https://github.com/nvalimak/fsm-lite) and unitig-caller (37), a k-mer is only defined as “present” in a genome when it shows an exact match in sequence content and sequence orientation. If this region is, for instance, inverted in other genomes, this k-mer is defined as “absent” in those genomes. This property of k-mer/unitig generation tools can be leveraged to detect rearranged genome sequences that are associated with phenotype of interest. Given the absence of single nucleotide polymorphisms and insertions-deletions, significant intact k-mers from bGWAS reflect rearranged genome sequences that are influenced by an inversion associated with a phenotype. This method is, however, not suitable for detecting translocations, as they do not involve any change in sequence orientation.

For significant k-mers that do not contain placeholder sequences, they are aligned with the genome sequences by BLAST using default parameters, as previously. The k-mers are then further filtered based on their BLAST alignment information using the same criteria 1-3 as previously but using an alternative criterion 4, whereby the k-mer should produce one unique BLAST alignment per genome. Then, for the k-mers passing the filters, summaries of count and proportion of case and control genomes containing the k-mers in forward/reverse orientation, as well as summary statistics of their genome positions are generated. They are available in the output file myNoNintactk_out.txt in /kmers_noN directory. Descriptions for each column are provided in the “Pipeline and output file description” section on GitHub.

BLAST results for k-mers with more than one hit per genome are also output for downstream investigation and the possibility of gene duplication (output file: kmer_with_multi_hits_noN.txt in in /kmers_noN directory).

9. Visualising genome rearrangements by plotting deduplicated k-mers/unitigs

For split k-mers containing placeholder sequences, rearrangements are visualised by plotting where the flanking sequences are found in case and control genomes. Due to the overlapping property of k-mers, multiple split k-mers can be found at a single rearrangement boundary. For output simplicity, these k-mers are deduplicated so that each rearrangement boundary is visualised by the plot of one k-mer. They are deduplicated as follows: each k-mer is given a label, which contains its: count and proportion of flanking sequences behaviour in case/control genomes, genome position (represented by the mean StartL when the k-mer is intact in control genomes, rounded off to two significant digits, as indicated by the -dedupk flag), and forward/reverse intact k-mers count. Only k-mers with unique labels are kept for visualisation. Deduplicated split k-mers can be found in output file myshort_splitk_out_uniq.txt in /kmers_withN directory. Additionally, since flanking sequences can show slightly different positions in different genomes due to insertions/deletions, this range of genome positions is shown by plotting out flanking sequences' positions in all genomes. To improve the clarity of presentation, flanking sequences (of the same colour) that are less than a certain number of base pairs apart (indicated by -dist flag, default is 40 000bp) are merged into one arrow by taking the median genome position value.

For intact k-mers with and without placeholder sequence as well as unitigs (denoted as k-mers), rearranged sequence contents are visualised by plotting where the k-mers are found in case and control genomes. Intact k-mers are deduplicated as follows: each k-mer is given a label, which contains its: median EndL when the k-mer is in forward orientation in control genomes, median EndL when the k-mer is in reverse orientation in control genomes, median EndL when the k-mer is in forward orientation in case genomes and median EndL when the k-mer is in reverse orientation in case genomes. For intact k-mers without placeholder sequence as well as unitigs, "sStart" values in BLAST output are used instead of EndL. After rounding off these values to the closest multiplier of selected value (*e.g.* 100, 1 000, 10 000), as indicated by the -intkrd flag, only k-mers with unique labels are kept for visualisation. The list of significant deduplicated k-mers that are used for visualisation can be found in files with suffix *kmer4plot.txt.

Figure S1: *GWarrange* pipeline summary chart.

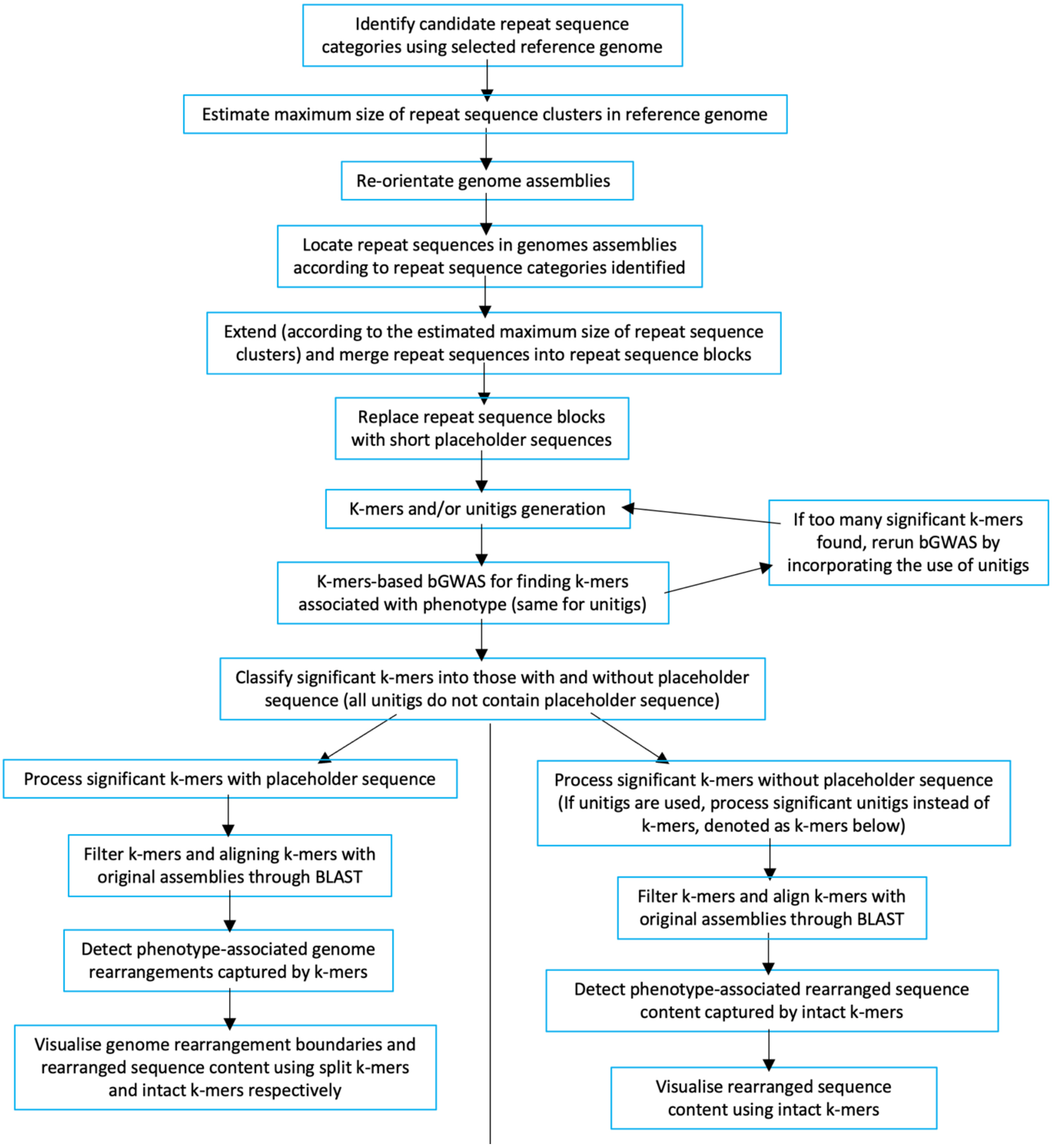

Figure S2: Start and end genome coordinates of left and right flanking sequences of a k-mer containing placeholder sequence.

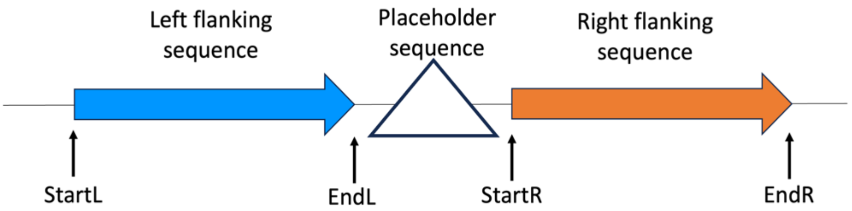

Figure S3: Schematic diagram of the two different genome structures explained by two nested inversions, present in a subset of 47 *B. pertussis* genomes. The outer inversion took place between genome coordinates 430 Kbp and 3600 Kbp, while the inner one took place between 1500 Kbp and 2500 Kbp, one inversion nested within the other.

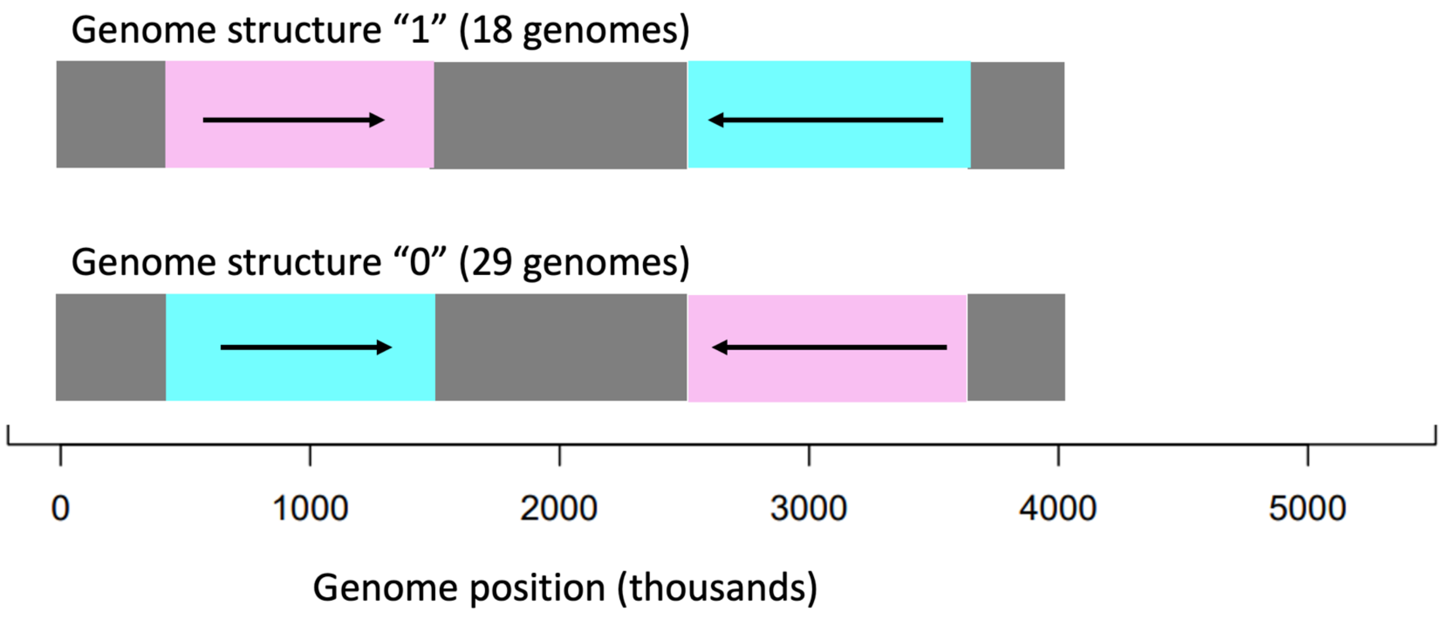

Figure S4: Example plots of four split k-mers that indicate inversion boundaries, from genome set with 7 000bp extension.

a) Inversion within genome between 430Kbp and 3600Kbp, 430Kbp boundary, k-mer being intact in majority of case genomes in reverse orientation and split in majority of control genomes
b) Inversion within genome between 430Kbp and 3600Kbp, 3600Kbp boundary, k-mer being intact in majority of control genomes in forward orientation and split in majority of case genomes
c) Inversion within genome between 1500Kbp and 2500Kbp, 1500Kbp boundary, k-mer being intact in majority of case genomes in reverse orientation and split in majority of control genomes
d) Inversion within genome between 1500Kbp and 2500Kbp, 2500Kbp boundary, k-mer being intact in majority of control genomes in forward orientation and split in majority of case genomes

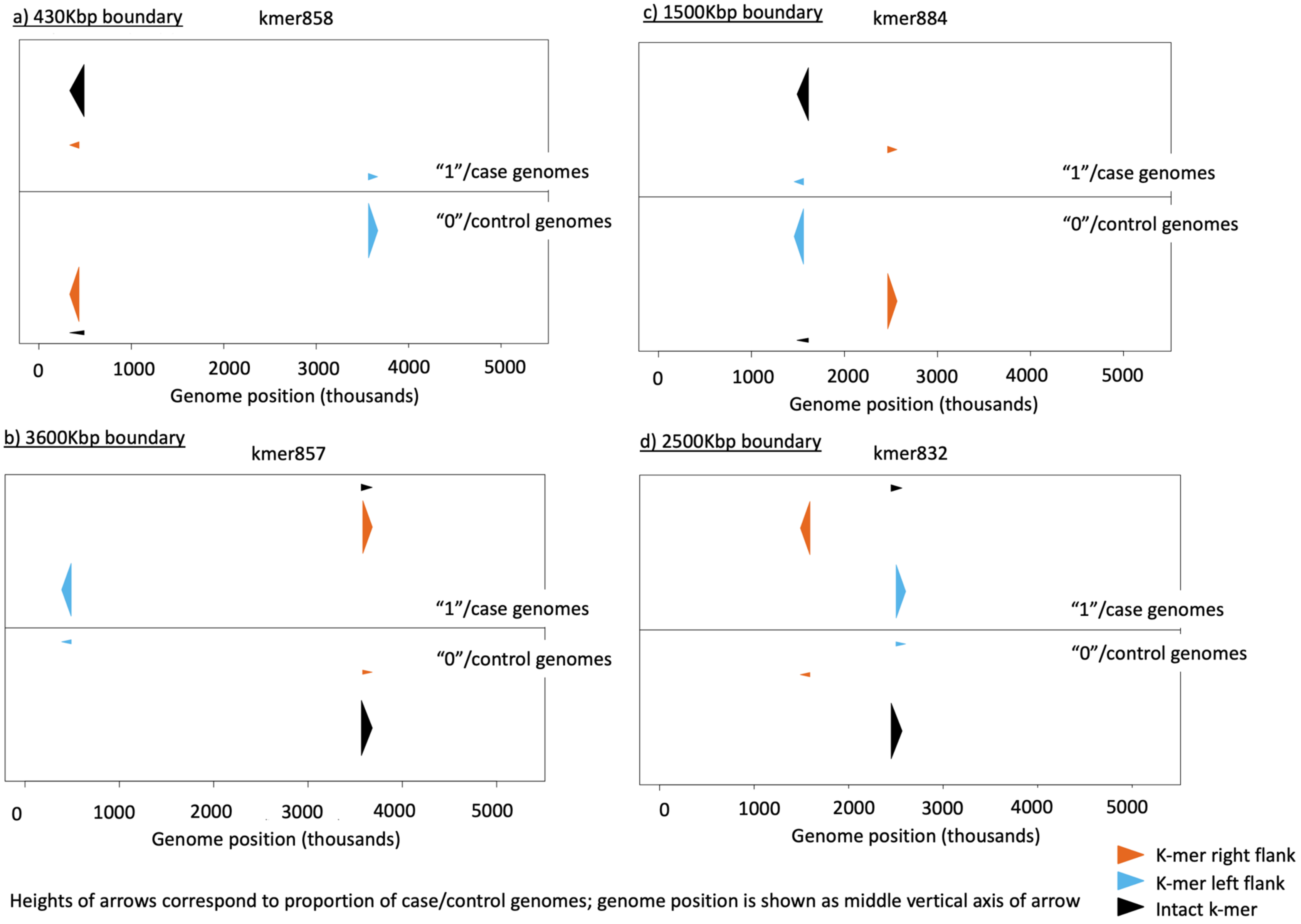

Heights of arrows correspond to proportion of case/control genomes; genome position is shown as middle vertical axis of arrow

Figure S5: Sixteen different significant split k-mers that were mapped to each of the inversion boundaries in example 1, split in case/control genomes, in forward/reverse orientation.

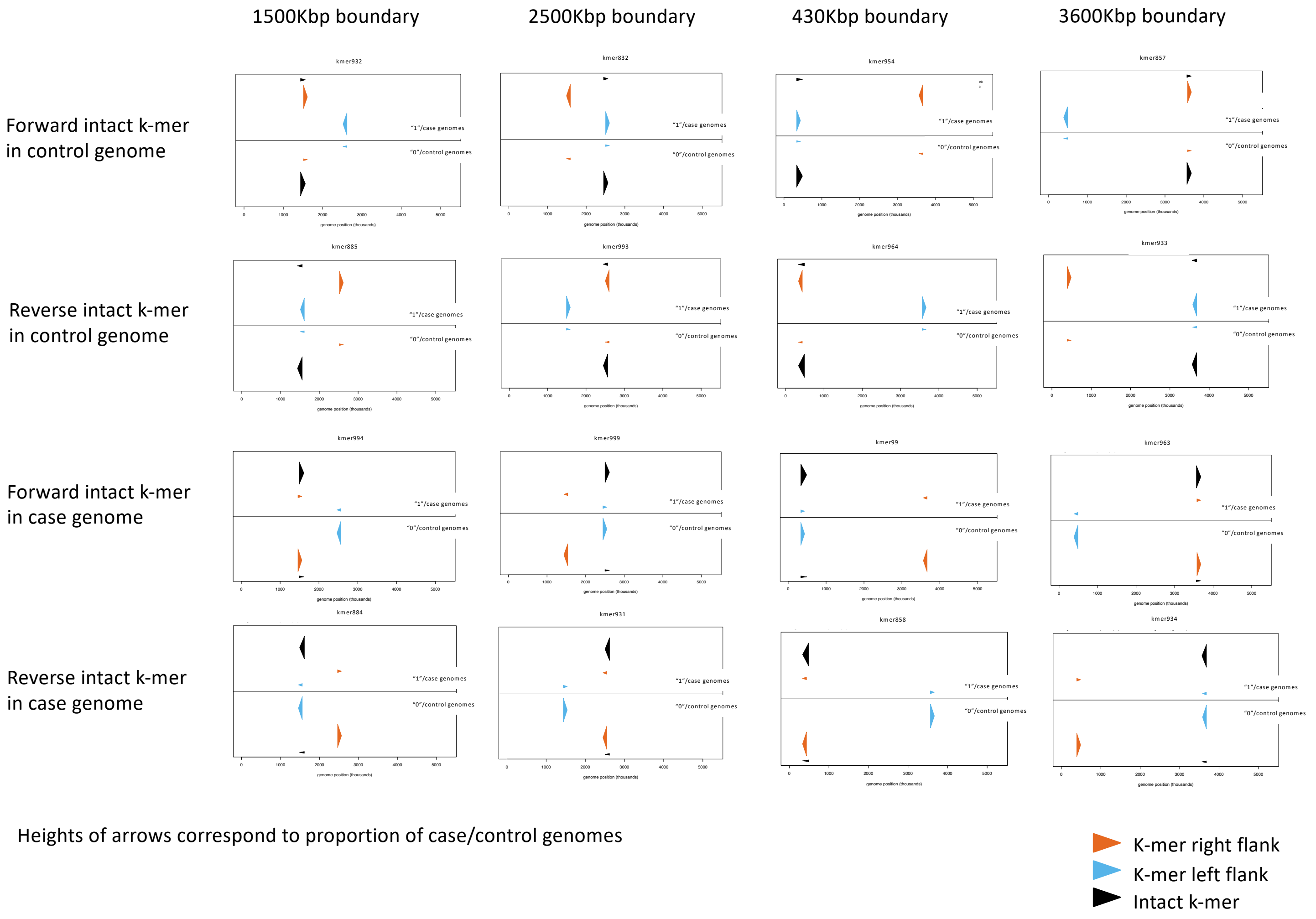

Figure S6: 1500 Kbp and 2500 Kbp boundaries in example 1 contain additional repeat sequences (highlighted in red) (*i.e.* peroxide stress protein YaaA, FUSC family membrane protein, MFS transporter) locating immediately adjacent to IS elements clusters (highlighted in green). MAUVE genome visualisation.

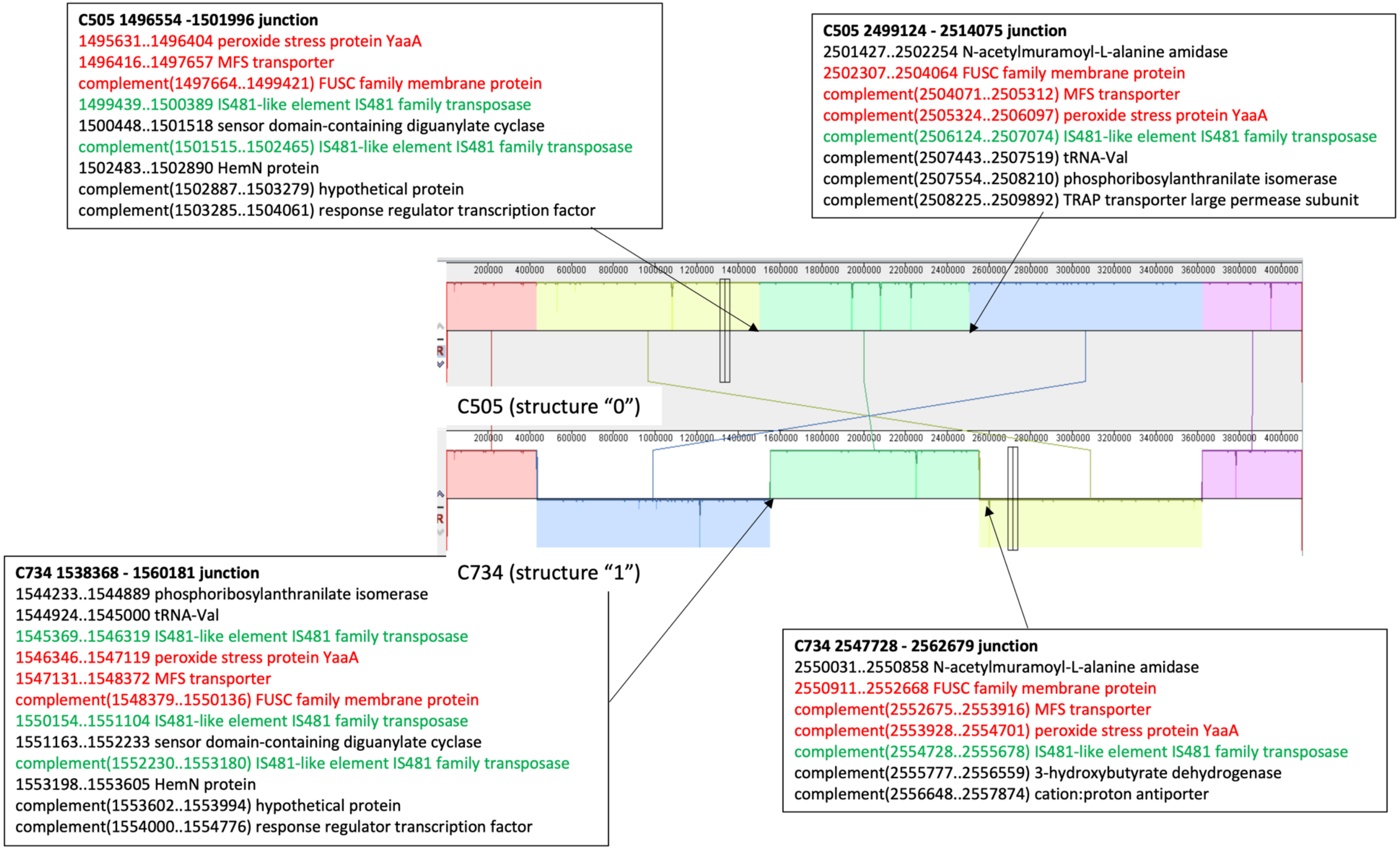

Figure S7: Plots of intact deduplicated k-mers (with and without placeholder sequence combined) visualise rearranged sequence content in two genome regions as a result of two nested inversions as depicted in Figure S3, significantly associated with genome structure phenotype. When using 100bp (a and b) and 7000bp (c and d) extension, and when intact k-mers are in forward (a and c) and reverse orientation (b and d) in majority of control genomes (genome phenotype group “0”). The direction of arrows indicates orientation of k-mers when mapped to genomes. Height of arrow corresponds to proportion of case (genome phenotype group “1”) / control (genome phenotype group "0”) genomes. Genome position of k-mer is indicated by the tip of arrow.

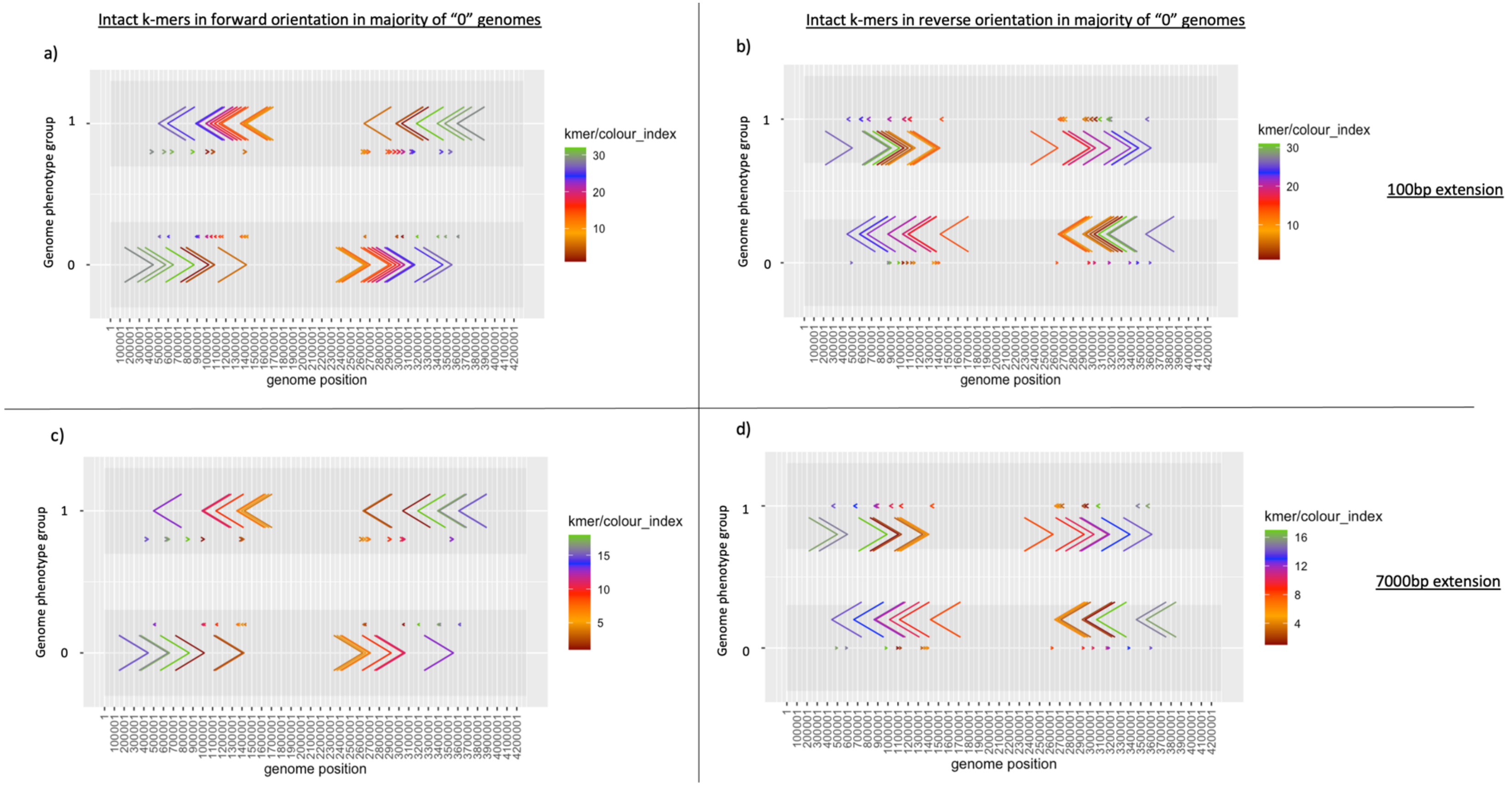

Height of arrows corresponds to proportion of case/control genomes
Genome positions of k-mers are indicated by the tips of arrows
K-mer/colour indices refer to the mycol_index column in the corresponding kmer4plot.txt

Figure S8: Schematic diagram of the two genome structures observed in 32 *E. faecium* genomes in example 2. The two structures are distinguished by an inversion.

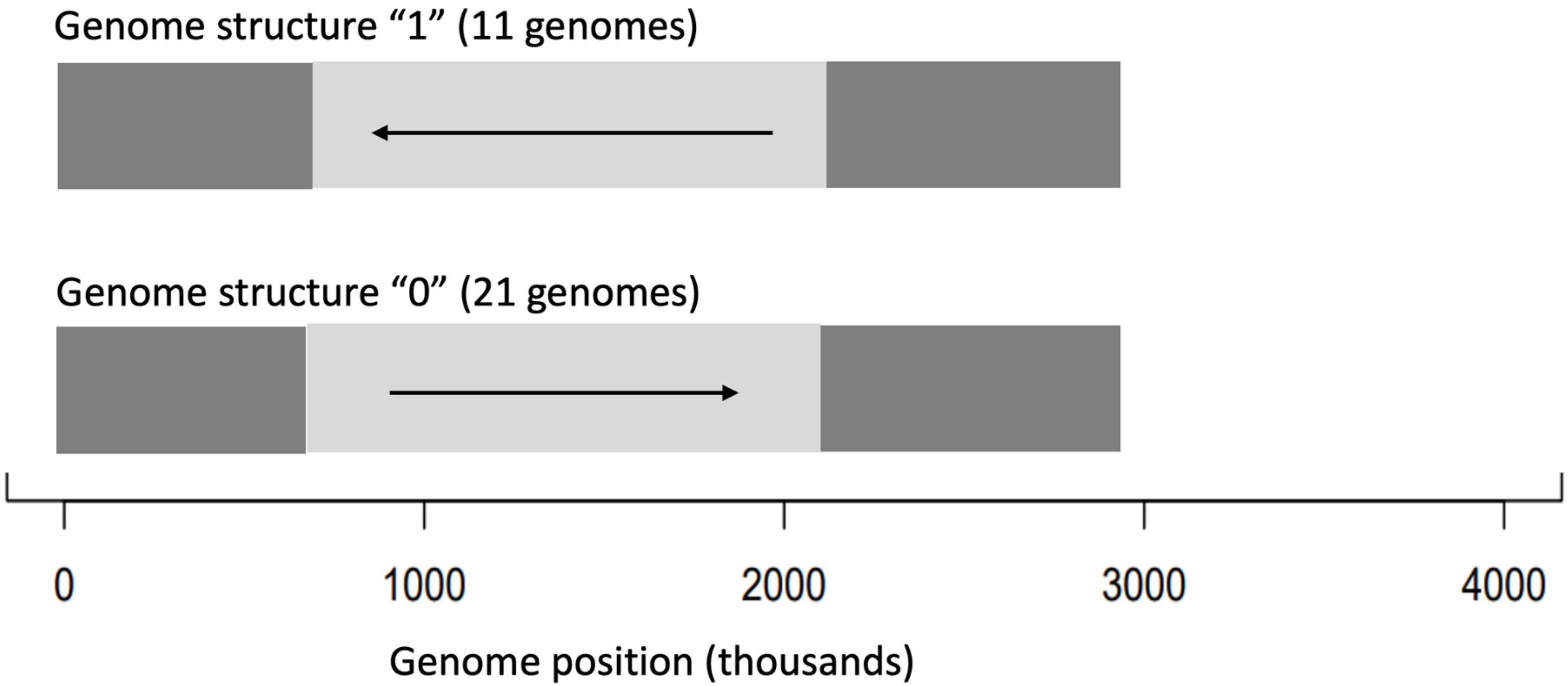

Figure S9: Plots of four split k-mers that indicate inversion boundaries at 720 Kbp and 2100 Kbp, generated using a 100bp extension.

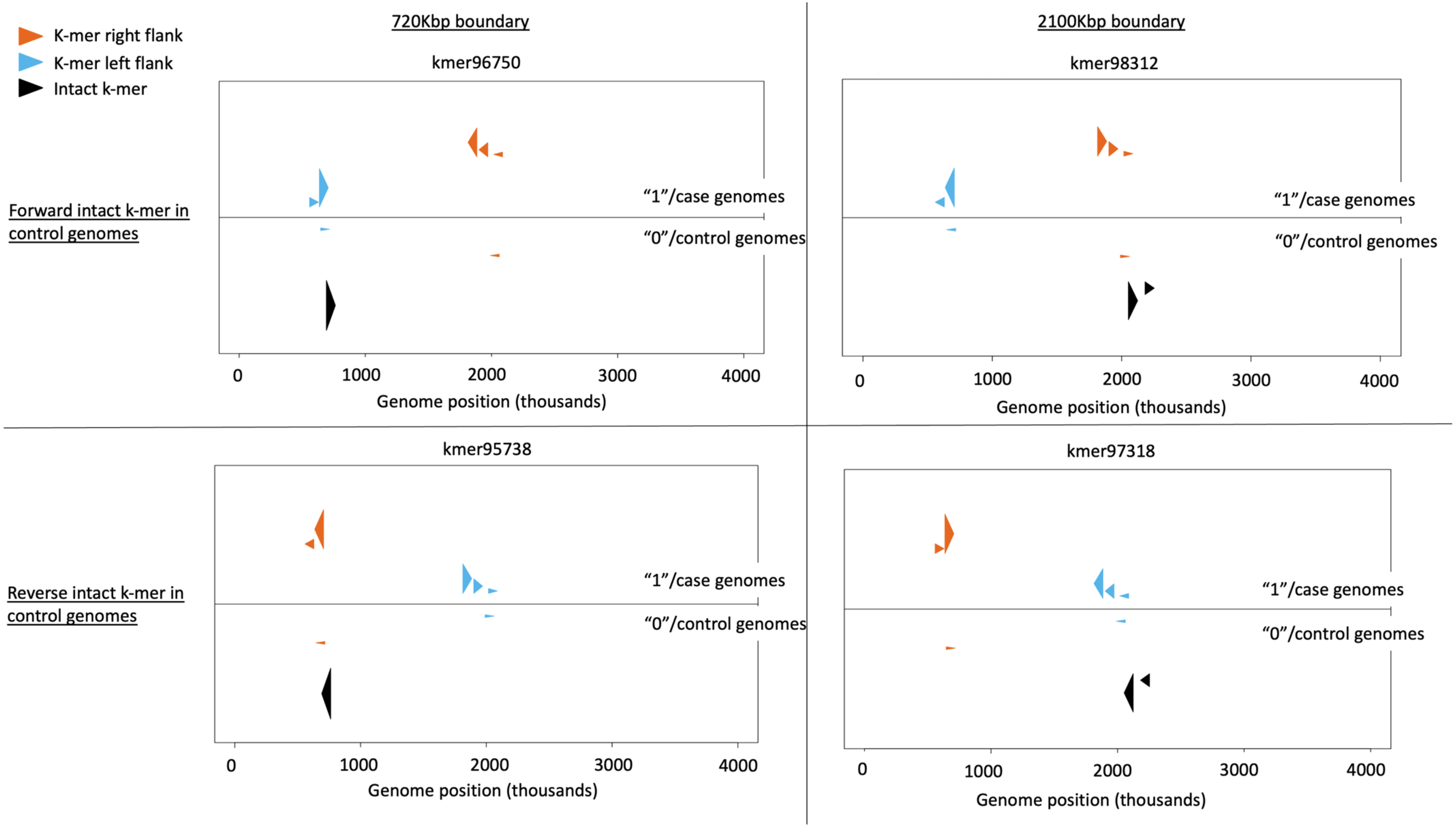

Heights of arrows correspond to proportion of case/control genomes; genome position is shown as middle vertical axis of arrow

Figure S10: Plots of deduplicated significant unitigs that showed rearranged sequence content significantly associated with predefined genome structure phenotype, when using 100bp (a and b) and 17000bp (c and d) extension, and when unitigs are in forward (a and c) and reverse orientation (b and d) in majority of control genomes. Height of arrow corresponds to the proportion of case (genome phenotype group “1”) /control (genome phenotype group “0”) genomes. Direction of arrows indicates orientation of unitigs when mapped to genomes. Genome position of unitigs indicated by the tip of arrow.

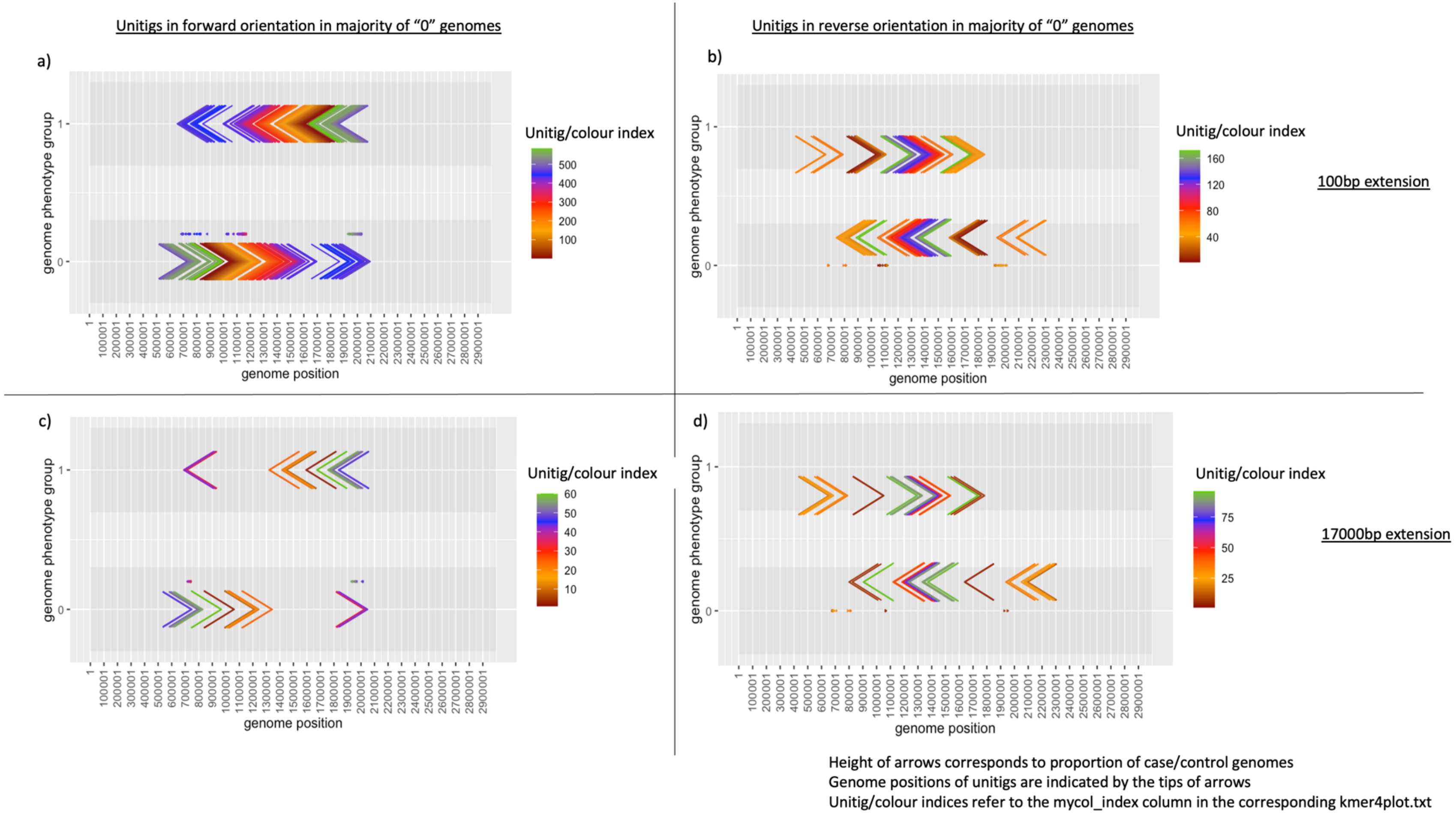

Figure S11: Schematic diagram for example 3 showing location of pertactin autotransporter gene is immediately next to a repeat region in the *B. pertussis* genomes J405 (accession: GCA_008817455.1), resulting it to be completely embedded within the region for replacement when 7000bp extension was applied.

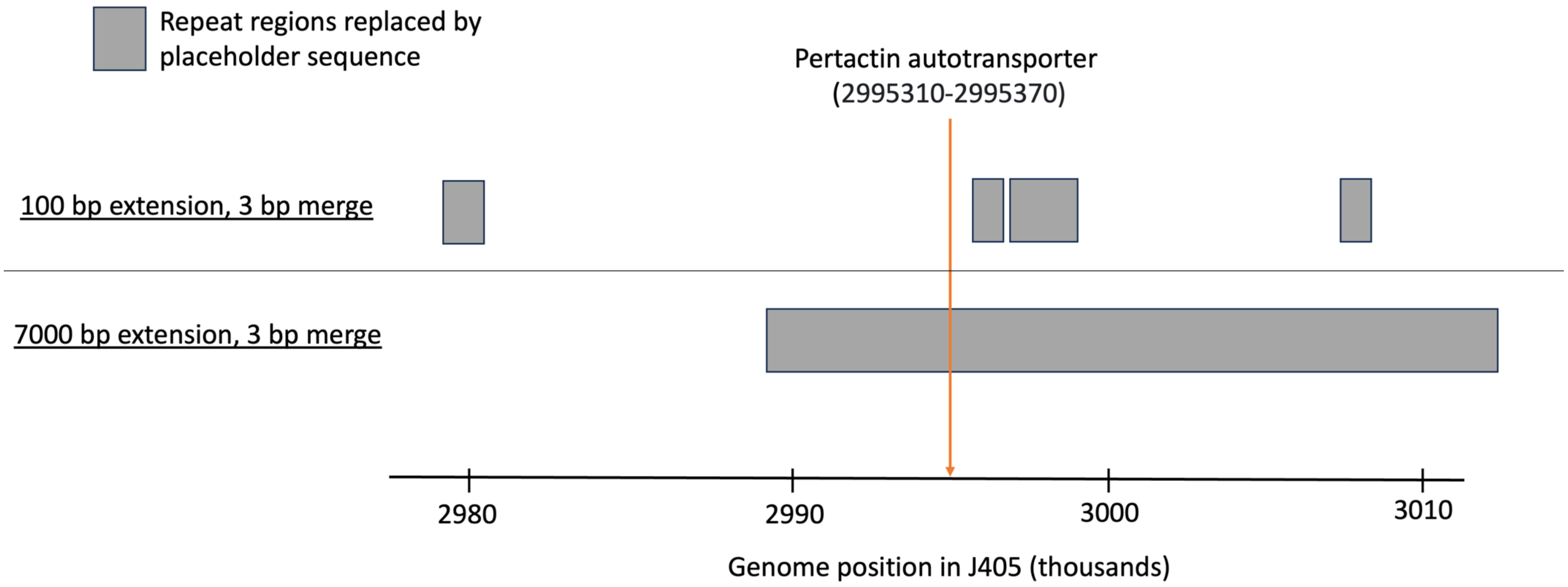

Figure S12: Schematic diagram of the two different genome structures explained by two translocation events, present in 39 simulated genomes (19 genomes with structure “0” and 20 genomes with structure “1”). Ribosomal operons that consist of mainly 16S ribosomal RNA, 23S ribosomal RNA, 5S ribosomal RNA and tRNAs are inserted in each of the rearrangement boundaries of the simulated genomes.

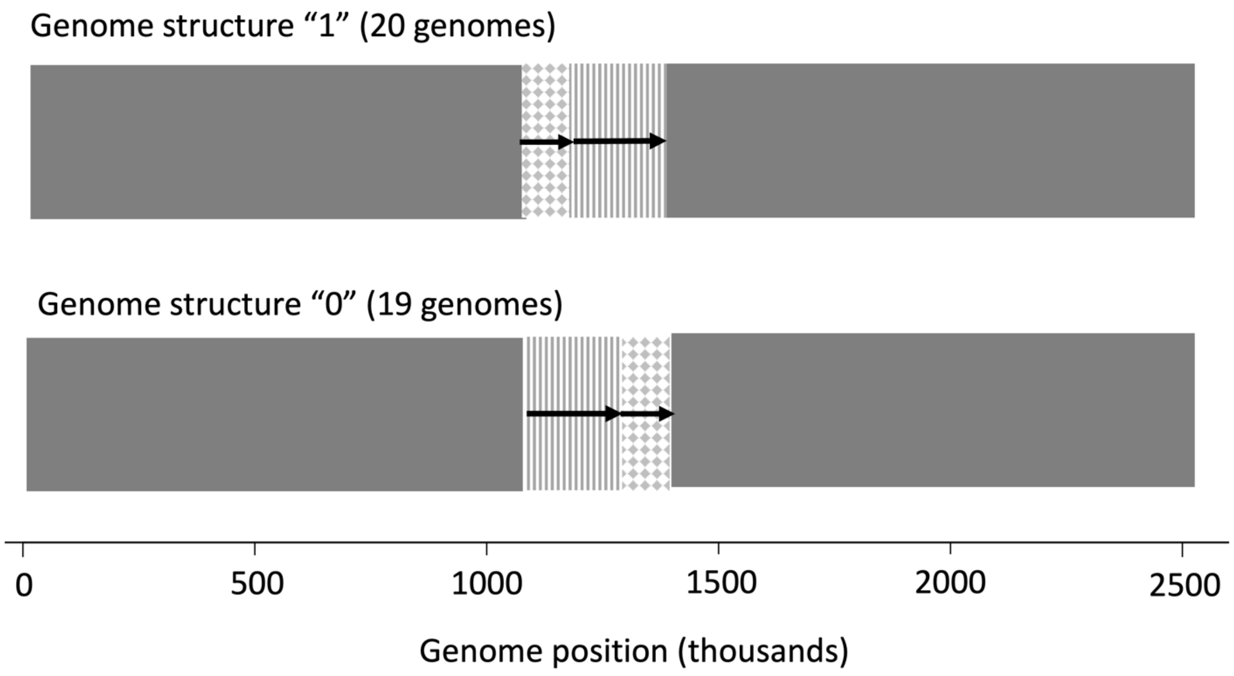

Figure S13: Twelve different significant split k-mers that were mapped to each of the translocation boundaries in example 4, split in case/control genomes, and in forward/reverse orientation.

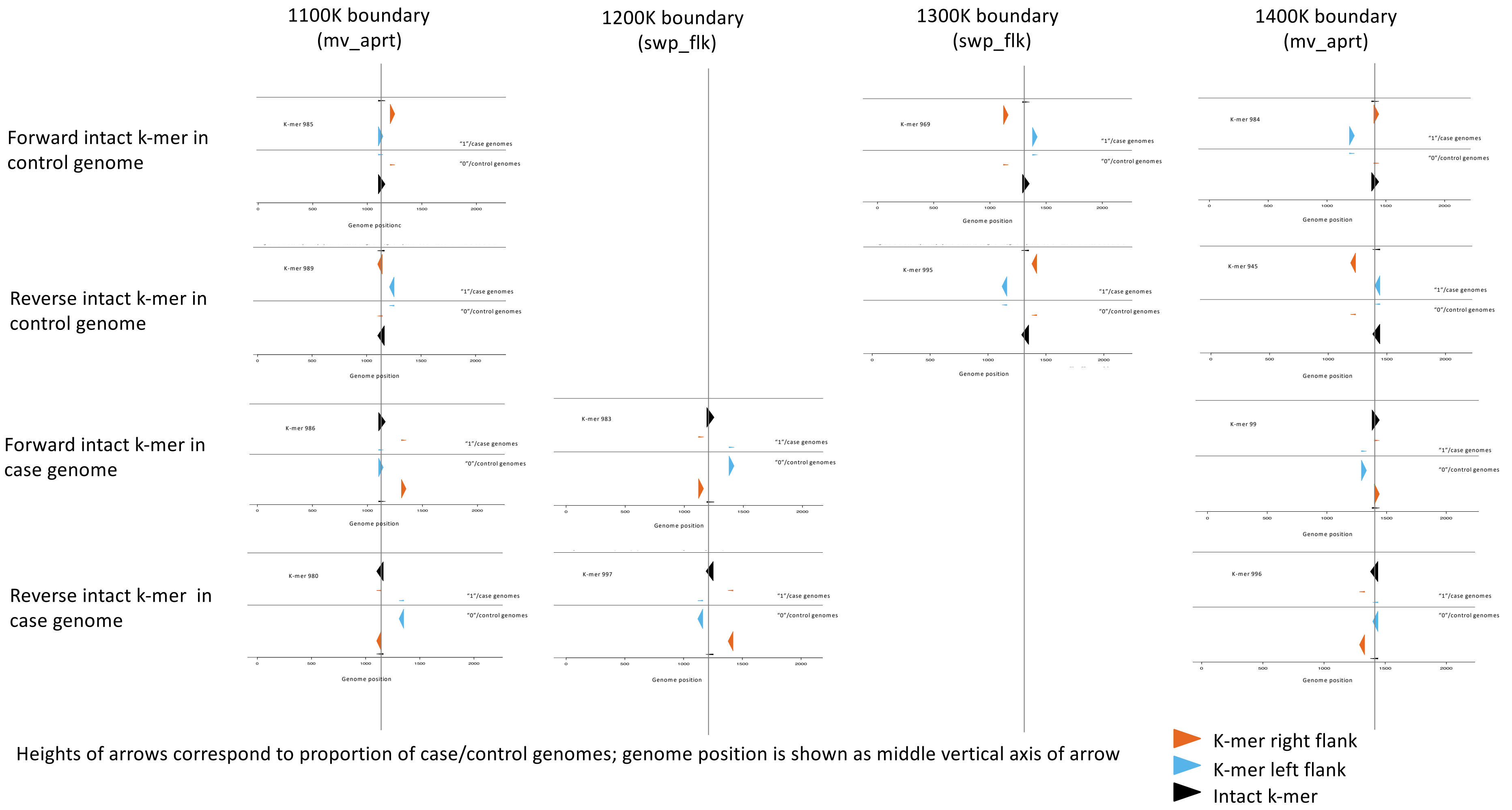

Heights of arrows correspond to proportion of case/control genomes; genome position is shown as middle vertical axis of arrow
